# The protocatechuic acid-based deep eutectic solvent-mediated green synthesis of 1,2,4,5-tetrasubstituted imidazoles †

**DOI:** 10.1039/d4ra03302g

**Published:** 2024-07-16

**Authors:** Hadis Goudarzi, Davood Habibi, Arezo Monem

**Affiliations:** a Department of Organic Chemistry, Faculty of Chemistry and Petroleum Sciences, Bu-Ali Sina University Hamedan 6517838683 Iran davood.habibi@gmail.com dhabibi@basu.ac.ir +98 81 31408025 +98 81 38380922

## Abstract

A novel protocatechuic acid-based deep eutectic solvent (ETPPBr/PCA-DES) was prepared by mixing ethyltriphenylphosphonium bromide (ETPPBr) and protocatechuic acid (PCA = 3,4-dihydroxybenzoic acid), and its structure was fully investigated by using the FT-IR, TGA/DTA, densitometer, eutectic point and ^1^H NMR techniques. Different molar ratios of ETPPBr to PCA were examined and the eutectic point phase diagram showed that the best ratio for the synthesis of the new DES is the one-to-one ratio of the two starting materials (ETPPBr and PCA). Then, the novel DES was used as a new and capable catalyst for the green synthesis of diverse 1,2,4,5-tetrasubstituted imidazoles a1–a12 from the four-component condensation reaction of phenanthrene-9,10-dione, aromatic amine, aromatic aldehyde, and ammonium acetate with high yields and very short reaction times. High yields and very short reaction times are two advantages of our proposed method compared with the previous reported methods.

## Abstract 2

ETPPBr/PCA-DES was prepared by mixing ETPPBr and PCA, and characterized by various techniques, and was used as a capable catalyst for the green synthesis of 1,2,4,5-tetrasubstituted imidazoles.

## Graphical abstract

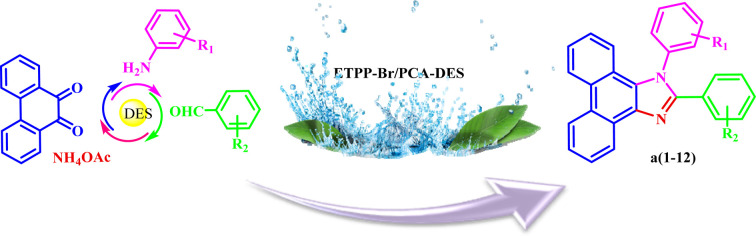

## 1. Introduction

In recent years, the use of new solvents such as ionic liquids (ILs) and deep eutectic solvents (DES) as powerful solvents with unique properties is of great importance in the synthesis of organic compounds. More recently, a new generation of green solvents, namely DESs, have emerged as a suitable alternative to hazardous organic solvents in various applications, and have been used as a low-cost alternative to ILs to overcome their disadvantages such as non-biodegradability or high toxicity.^1^ But some scientists consider them to be a new class of ILs because they have similar properties to ILs.^2^ The basis of the formation of DESs is the formation of hydrogen bonds between two or more hydrogen bond donor (HBD) and hydrogen bond acceptor (HBA) components, which results in a mixture with a lower melting point than their constituents.^3,4^ A DES generally consists of two or three safe and inexpensive components that are linked together through hydrogen bonding interactions and form a eutectic mixture.^5^ In DESs, the hydrogen bond acceptor component is usually phosphonium or ammonium salt, and the hydrogen bond donor component is alcohol, acid, amine, amino acid, *etc.*^6–9^ In these compounds, the unusual reduction of the melting point of the eutectic mixture is significant compared to the ingredients. This behavior was reported for the first time in the combination of 1 : 2 molar fraction of choline chloride powder with a melting point of 302 °C and urea crystal with a melting point of 133 °C, which led to the formation of a eutectic mixture with a melting point of 12 °C.^10^ The use of DES for the synthesis of organic compounds in catalyzed and uncatalyzed reactions is considered one of their important applications. Among the reactions carried out in this field: substitution, condensation, oxidation and reduction reactions,^11^ esterification and halogenation reactions,^12^ synthesis of pyridine derivatives,^13^ synthesis of 5-ethoxymethylfurfural,^14^ synthesis of piperidine derivatives^15^ and so on.

Multicomponent reactions (MCRs) involve more than two chemicals in a one-pot process that leads to the production of a specific product. In ordinary multi-step syntheses, a lot of waste is usually produced and it is difficult to separate each batch, so MCRs are important from an environmental and economic point of view.^16,17^

Polycyclic heterocyclic molecules are significant as an important group of organic materials in the field of optical materials, medicine, and sensors.^18–20^ The first synthesis route to the imidazole nucleus was reported in 1882 (ref. 21 and 22) and the name imidazole was first coined by a German chemist in 1887.^23^ Poly-substituted imidazoles can potentially have new therapeutic activities and in recent years, due to their many chemical and biological applications, they have attracted a lot of attention in the field of industry and pharmaceuticals. The biological and medicinal applications of poly-substituted imidazoles are: anti-cancer,^24^ anti-inflammatory,^25^ anti-tumor,^26^ antibacterial,^27^ non-nucleoside inhibitor against HIV-1 reverse transcriptase,^28^ antidiabetic,^29^ anticonvulsant,^30^ and treatment of tuberculosis.^31^

**Scheme 1:**
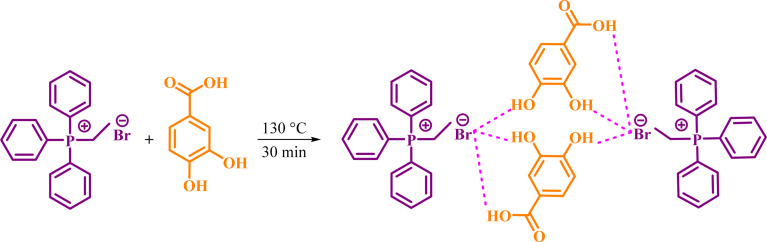
Synthesis of the novel DES.

In continuing our researches to find new catalysts, we would like to report preparation of a novel DES from the one-to-one molar ratio of ETPPBr (ethyltriphenylphosphonium bromide) and PCA (protocatechuic acid) (Scheme 1).

**Scheme 2:**
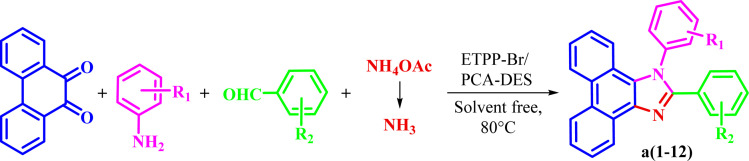
Synthesis of 1,2,4,5-tetrasubstituted imidazoles.

Then, the prepared novel DES was used as a new and capable catalyst for the green synthesis of 1,2,4,5-tetrasubstituted imidazoles in the four component condensation reactions of phenanthrene-9,10-dione, aromatic amine, aromatic aldehyde, and ammonium acetate in solvent-free conditions at 80 °C (Scheme 2).

## 2. Experimental

### 2.1. Materials

Chemicals were purchased from the Merck and Sigma-Aldrich companies. Progress of the reactions was monitored by thin layer chromatography (TLC) using silica gel 60-F-254 plates. Melting points were measured in capillary tubes using Stuart melting point instrument without correction. FT-IR spectra were recorded with KBr pellets on PerkinElmer Spectrometer. The NMR spectra were recorded on a Bruker Ultrashield 250 MHz spectrometer in CDCl_3_.

### 2.2. General procedure for preparation of ETPPBr/PCA-DES

The one-to-one molar ratio mixture of ETPPBr (m.p.: 206 °C) and PCA (m.p.: 221 °C) was heated at 130 °C and stirred until a homogeneous transparent liquid was obtained (about 30 to 40 minutes). It was cooled in room temperature and kept for further reactions. The melting point of the obtained novel DES is about 95–100 °C.

### 2.3. General procedure for the synthesis of a1–a12

A mixture of phenanthrene-9,10-dione (1 mmol), aromatic amine (1 mmol), aromatic aldehyde (1 mmol), ammonium acetate (1.5 mmol) and the DES catalyst (0.5 g) was heated and stirred at 80 °C for appropriate times under solvent-free conditions (Table 4). The progress of the reaction was monitored (a sample is removed from the reaction using a spatula and dissolved in acetone) by TLC (ethyl acetate/*n*-hexane 2 : 8). Also, the spots of the products are bright and fluorescent which is a good help to check the reaction progress.

After completion of the reaction (TLC), ethanol (10 mL) was added to the mixture, filtered, washed by hot ethanol, dried, and characterized with comparison of their FT-IR, ^1^H NMR, ^13^C NMR, Mass spectra, and melting points with authentic samples.

The mixture of chloroform/water (50 : 50, 20 mL) was added to ethanolic filtrate, decanted, the DES catalyst was separated from the aqueous phase, dried, and stored for further reactions.

#### 2.3.1. 2-(3-Nitrophenyl)-1-phenyl-1*H*-phenanthro[9,10-*d*]imidazole (a1)

Green solid, M.P.: 206–208 °C; IR (KBr) *ν* = 3057, 1619, 1524, 1497, 1168, 1045, 754, 669 cm^−1^; ^1^H NMR (250 MHz, CDCl_3_) *δ* = 8.86 (d, *J* = 7.8 Hz, 1H), 8.72 (dd, *J* = 16.2, 8.3 Hz, 2H), 8.36 (s, 1H), 8.12 (d, *J* = 8.1 Hz, 1H), 8.00 (d, *J* = 7.8 Hz, 1H), 7.75 (t, *J* = 7.3 Hz, 2H), 7.67 (d, *J* = 6.9 Hz, 3H), 7.60–7.37 (m, 4H), 7.34–7.17 (m, 2H). ^13^C NMR (62.5 MHz, CDCl_3_) *δ* = 134.84, 130.61, 129.24, 128.85, 127.47, 126.46, 126.00, 125.40, 124.17, 123.79, 123.24, 122.73, 120.91, 77.53, 77.03, 76.52. MS: *m*/*z* = 415 [M^+^], 415 (base peak).

#### 2.3.2. 1-(4-Chlorophenyl)-2-(4-isopropylphenyl)-1*H*-phenanthro[9,10-*d*]imidazole (a2)

White solid, M.P.: 234–236 °C; IR (KBr) *ν* = 3050, 2956, 2869, 1611, 1573, 1492, 1471, 1375, 1084, 845, 754, 669 cm^−1^; ^1^H NMR (250 MHz, CDCl_3_) *δ* = 8.88 (d, *J* = 7.8 Hz, 1H), 8.73 (dd, *J* = 17.7, 8.2 Hz, 2H), 7.60 (ddt, *J* = 46.9, 21.2, 7.3 Hz, 9H), 7.30 (q, *J* = 6.3 Hz, 2H), 7.17 (s, 2H), 2.90 (p, *J* = 6.8 Hz, 1H), 1.24 (d, *J* = 6.8 Hz, 6H). ^13^C NMR (62.5 MHz, CDCl_3_) *δ* = 151.07, 150.03, 137.31, 135.71, 130.43, 129.38, 127.35, 126.99, 126.51, 126.36, 125.73, 124.95, 124.21, 123.08, 122.86, 120.60, 77.53, 77.03, 76.52, 33.91, 23.74. MS: *m*/*z* = 446 [M^+^], 446 (base peak).

#### 2.3.3. 1-(4-Chlorophenyl)-2-(3-nitrophenyl)-1*H*-phenanthro[9,10-*d*]imidazole (a3)

Yellow solid, M.P.: 182–185 °C; IR (KBr) *ν* = 3093, 1609, 1581, 1526, 1342, 1088, 1018, 761, 706 cm^−1^; ^1^H NMR (250 MHz, CDCl_3_) *δ* = 8.89–8.65 (m, 3H), 8.42 (s, 1H), 8.17 (d, *J* = 8.3 Hz, 1H), 7.92 (d, *J* = 7.8 Hz, 1H), 7.83–7.60 (m, 4H), 7.60–7.42 (m, 4H), 7.40–7.19 (m, 2H). ^13^C NMR (62.5 MHz, CDCl_3_) *δ* = 147.94, 136.58, 134.78, 131.69, 130.89, 130.20, 129.46, 128.46, 127.59, 126.65, 126.19, 125.60, 124.31, 124.05, 123.55, 123.19, 122.76, 120.70, 77.52, 77.01, 76.51. MS: *m*/*z* = 449 [M^+^], 449 (base peak).

#### 2.3.4. 1-(4-Chlorophenyl)-2-(*p*-tolyl)-1*H*-phenanthro[9,10-*d*]imidazole (a4)

Cream solid, M.P.: 181–182 °C; IR (KBr) *ν* = 3045, 2910, 1611, 1575, 1493, 1379, 1090, 1018, 753, 673 cm^−1^; ^1^H NMR (250 MHz, CDCl_3_) *δ* = 8.87 (d, *J* = 7.8 Hz, 1H), 8.73 (dd, *J* = 17.7, 8.2 Hz, 2H), 7.70 (dt, *J* = 22.5, 7.0 Hz, 3H), 7.56 (d, *J* = 7.9 Hz, 1H), 7.52 (s, 1H), 7.43 (d, *J* = 7.8 Hz, 4H), 7.38–7.18 (m, 2H), 7.13 (d, *J* = 7.6 Hz, 2H), 2.35 (s, 3H). ^13^C NMR (62.5 MHz, CDCl_3_) *δ* = 151.09, 139.26, 137.23, 135.72, 130.42, 129.39, 129.10, 128.29, 127.37, 126.93, 126.40, 125.77, 125.01, 124.22, 123.09, 122.89, 120.62, 77.55, 77.04, 76.54, 21.33. MS: *m*/*z* = 418 [M+], 418 (base peak).

#### 2.3.5. 2-(4-Isopropylphenyl)-1-phenyl-1*H*-phenanthro[9,10-*d*]imidazole (a5)

White solid, M.P.: 188–189 °C; IR (KBr) *ν* = 3063, 2960, 1609, 1597, 1496, 1475, 849, 747, 698 cm^−1^; ^1^H NMR (250 MHz, CDCl_3_) *δ* = 8.91 (d, *J* = 7.9 Hz, 1H), 8.74 (dd, *J* = 15.7, 8.3 Hz, 2H), 7.81–7.64 (m, 2H), 7.63 (d, *J* = 2.0 Hz, 1H), 7.62–7.45 (m, 6H), 7.25 (d, *J* = 7.9 Hz, 2H), 7.20–7.10 (m, 3H), 2.88 (p, *J* = 7.0 Hz, 1H), 1.24 (s, 3H), 1.21 (s, 3H). ^13^C NMR (62.5 MHz, CDCl_3_) *δ* = 150.82, 138.61, 130.18, 129.88, 129.43, 129.13, 127.87, 127.38, 126.41, 126.29, 125.75, 124.95, 124.10, 123.07, 120.83, 77.54, 77.03, 76.52, 33.90, 23.72. MS: *m*/*z* = 412 [M^+^], 412 (base peak).

#### 2.3.6. 1,2-Diphenyl-1*H*-phenanthro[9,10-*d*]imidazole (a6)

White solid, M.P.: 200–202 °C; IR (KBr) *ν* = 3057, 1619, 1524, 1497, 1168, 1045, 754, 669 cm^−1^.

#### 2.3.7. 1-(4-Chlorophenyl)-2-(3,4-dimethoxyphenyl)-1*H*-phenanthro[9,10-*d*]imidazole (a7)

White solid, M.P.: 184–186 °C; IR (KBr) *ν* = 3052, 2991, 2836, 1601, 1568, 1494, 1260, 1140, 1022, 756, 725 cm^−1^.

#### 2.3.8. 2-(4-Chlorophenyl)-1-phenyl-1*H*-phenanthro[9,10-*d*]imidazole (a8)

Cream solid, M.P.: 234–235 °C; IR (KBr) *ν* = 3063, 1619, 1596, 1498, 1145, 1013, 757, 695 cm^−1^; ^1^H NMR (250 MHz, CDCl_3_) *δ* = 8.86 (d, *J* = 8.1 Hz, 1H), 8.72 (dd, *J* = 15.1, 8.4 Hz, 2H), 7.83–7.62 (m, 4H), 7.62–7.44 (m, 6H), 7.31 (d, *J* = 8.9 Hz, 1H), 7.29–7.14 (m, 3H).

#### 2.3.9. 1-Phenyl-2-(*p*-tolyl)-1*H*-phenanthro[9,10-*d*]imidazole (a9)

Cream solid, M.P.: 200–201 °C; IR (KBr) *ν*: 3063, 2907, 1619, 1597, 1495, 1187, 1037, 752, 696 cm^−1^; ^1^H NMR (250 MHz, CDCl_3_) *δ* = 8.74 (dd, *J* = 15.3, 8.3 Hz, 2H), 7.82–7.54 (m, 6H), 7.54–7.43 (m, 5H), 7.33–7.05 (m, 4H), 2.33 (s, 3H).

#### 2.3.10. 1-(4-Chlorophenyl)-2-phenyl-1*H*-phenanthro[9,10-*d*]imidazole (a10)

Cream solid, M.P.: 220–221 °C; IR (KBr) *ν* = 3055, 1609, 1572, 1494, 1089, 755, 698 cm^−1^.

#### 2.3.11. 1-(4-Chlorophenyl)-2-(4-methoxyphenyl)-1*H*-phenanthro[9,10-*d*]imidazole (a11)

Cream solid, M.P.: 240–244 °C; IR (KBr) *ν* = 3065, 2836, 1609, 1580, 1493, 1249, 1177, 1025, 760, 666 cm^−1^; ^1^H NMR (250 MHz, CDCl_3_) *δ* = 8.86 (d, *J* = 7.9 Hz, 1H), 8.72 (dd, *J* = 17.0, 8.3 Hz, 2H), 7.69 (dq, *J* = 15.1, 7.3 Hz, 2H), 7.58–7.41 (m, 6H), 7.38 (s, 1H), 7.34–7.11 (m, 2H), 6.84 (d, *J* = 8.4 Hz, 2H), 3.79 (d, *J* = 8.5 Hz, 3H). ^13^C NMR (62.5 MHz, CDCl_3_) *δ* = 160.27, 150.89, 137.27, 135.81, 130.93, 130.44, 129.49, 129.20, 128.25, 127.34, 126.83, 126.39, 125.74, 124.95, 124.19, 123.87, 123.07, 122.87, 122.25, 120.56, 113.85, 77.56, 77.05, 76.55, 55.26.

#### 2.3.12. 4-(1-(4-Chlorophenyl)-1*H*-phenanthro[9,10-*d*]imidazole-2-yl)-*N*,*N*-dimethylaniline (a12)

White solid, M.P.: 239–240 °C; IR (KBr) *ν* = 3086, 2984, 2803, 1607, 1480, 1361, 1196, 758, 66 cm^−1^.

## 3. Results and discussion

### 3.1. Characterization of ETPPBr/PCA-DES

The prepared novel DES compound was characterized by FT-IR, NMR, densitometer, eutectic point, and TGA-DTA techniques.

#### 3.1.1. Characterization by FT-IR

**Fig. 1:**
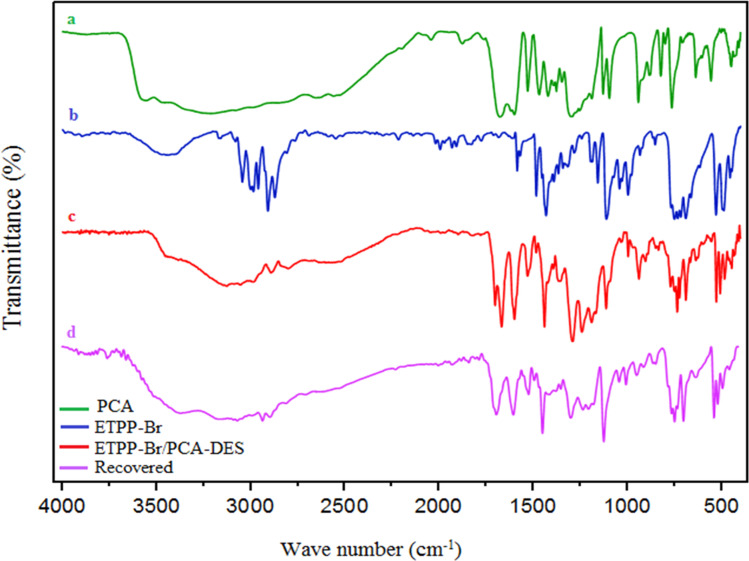
The FT-IR spectra of (a), (b), (c), and (d).

Fig. 1 shows the IR spectra of PCA (a), ETPPBr (b), the fresh DES (c), and the recovered DES (d). In spectrum (a), the peaks at 3600 and 1670 cm^−1^ are related to the O–H, and the C=O of the –COOH groups, respectively. In spectrum (b), the peaks at about 2900–3100 cm^−1^ are related to the aromatic and aliphatic hydrogens, and the peak at about 750 cm^−1^ is related to the C–P bonds, respectively. In the (c) spectrum, the mentioned peaks can be seen in both (a) and (b) spectra, which confirm the structure of the DES catalyst.

Also, the IR spectrum of the recovered DES was recorded to be compared with the fresh one. This shows that there is no significant difference between the fresh and recovered (used) IR spectra, and is another confirmation of the synthesized DES structure.

#### 3.1.2. Characterization by eutectic points

**Fig. 2:**
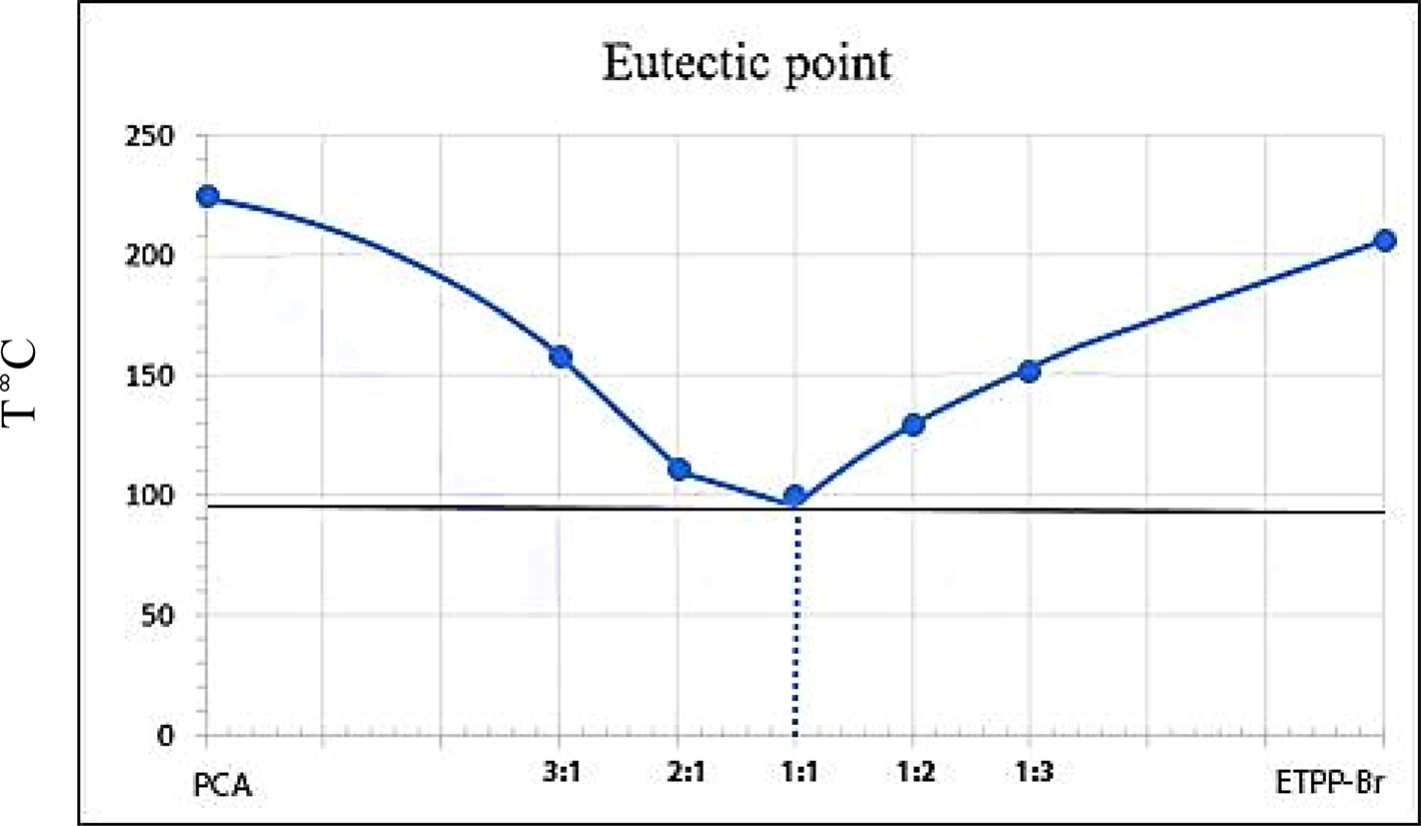
The eutectic point phase diagram of the novel DES.

Eutectic point test was performed to check the best ratio of ETPPBr to PCA. In this experiment, five different molar ratios of ETPPBr to PCA (3 : 1, 2 : 1, 1 : 1, 1 : 2 and 1 : 3) were obtained, and the melting point of the resulting mixture was measured in each step. The following phase diagram was obtained which shows that the best ratio of ETPPBr to PCA is one to one with melting point about 95–100 °C (Fig. 2).

#### 3.1.3. Characterization by ^1^H NMR

##### 3.1.3.1. The ^1^H NMR spectrum of ETPPBr

**Fig. 3:**
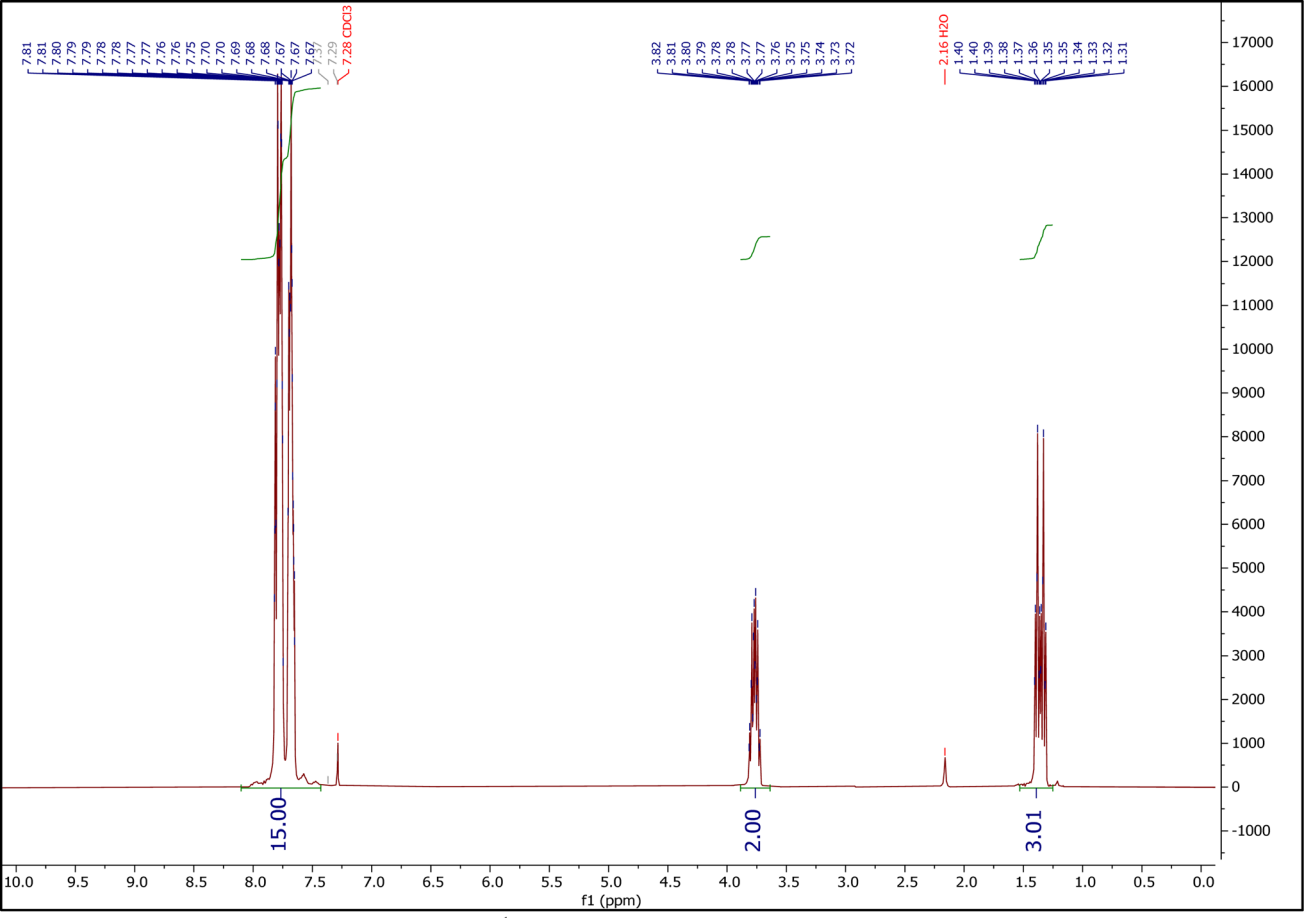
The ^1^H NMR spectrum of ETPPBr.

Fig. 3 shows the ^1^H NMR spectrum of ETPPBr. Peaks at 1.31–1.40 (integration = 3), 3.72–3.81 (integration = 2) and 7.65–7.82 (integration = 14.86) ppm are related to the CH_3_ hydrogens, CH_2_ hydrogens, and fifteen hydrogens of phenyl rings, respectively.

##### 3.1.3.2. The ^1^H NMR spectrum of PCA

**Fig. 4:**
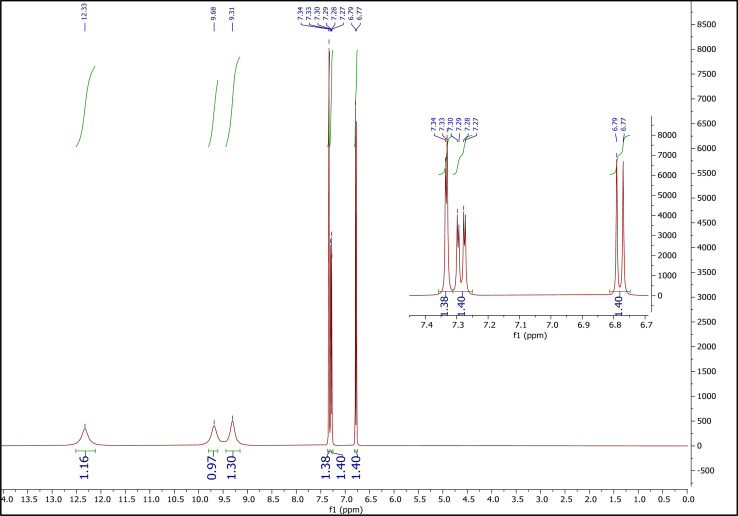
The ^1^H NMR spectrum of PCA.

Fig. 4 shows the ^1^H NMR spectrum of PCA. Peaks at 6.77–6.79 (integration = 1.40), 7.27–7.30 (integration = 1.40), and 7.33–7.34 (integration = 1.38), correspond to the hydrogens of phenyl ring. Peaks at 9.31 (integration = 1.30), and 9.68 (integration = 0.97) correspond to two hydrogens of two hydroxy groups and peak at 12.33 (integration = 1.16) is related to the hydrogen of the –COOH group.

##### 3.1.3.3. The ^1^H NMR spectrum of ETPPBr/PCA-DES

**Fig. 5:**
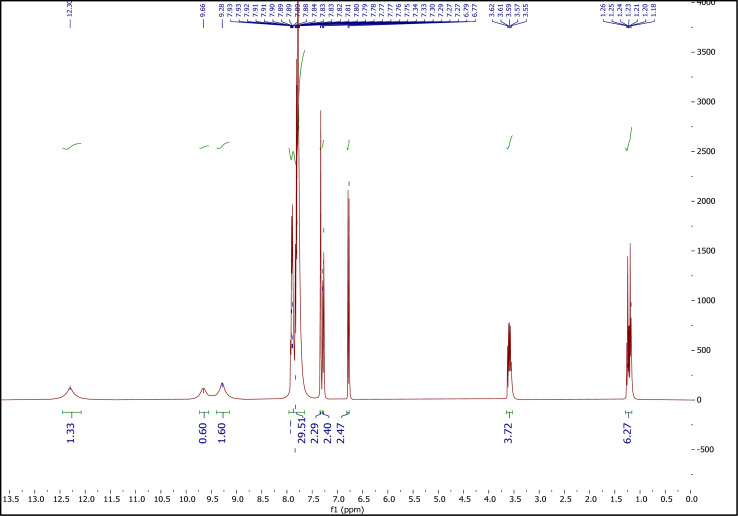
The ^1^H NMR spectrum of the novel DES.

Fig. 5 shows the ^1^H NMR spectrum of ETPPBr/PCA-DES. Peaks at 1.18–1.26 (6H), 3.55–3.62 (4H), 6.77–6.79 (2H), 7.27–7.30 (2H), 7.33–7.34 (1H), 7.75–7.93 (30H), 9.28 (2H), 9.66 (1H) and 12.30 (1H) are related to six hydrogens of two CH_3_ groups, four hydrogens of two CH_2_ groups, six hydrogens of two benzene rings of PCA, thirty hydrogens of six phenyl groups of ETPPBr, hydrogen of hydroxy group at position 4, hydrogen of hydroxy group at position 3 and hydrogen of the –COOH group, respectively.

The shift of the peaks of the ethyl group of ETPPBr to a lower place compared to the starting materials, that is, a shift from 1.31–1.40 to 1.18–1.26 (CH_3_) and from 3.72–3.82 to 3.55–3.62 (CH_2_), as well as a decrease in the intensity of the hydrogen signals are indications of the presence of hydrogen bonds and formation of the new DES.

Incidentally, a decrease in splitting patterns of the peaks in DES, compared to the starting materials can also be a very nice evidence of hydrogen bonds formation between the two components (ETPPBr and PCA).

##### 3.1.3.4. Comparison of the ^1^H NMR spectrum of PCA and DES

**Fig. 6:**
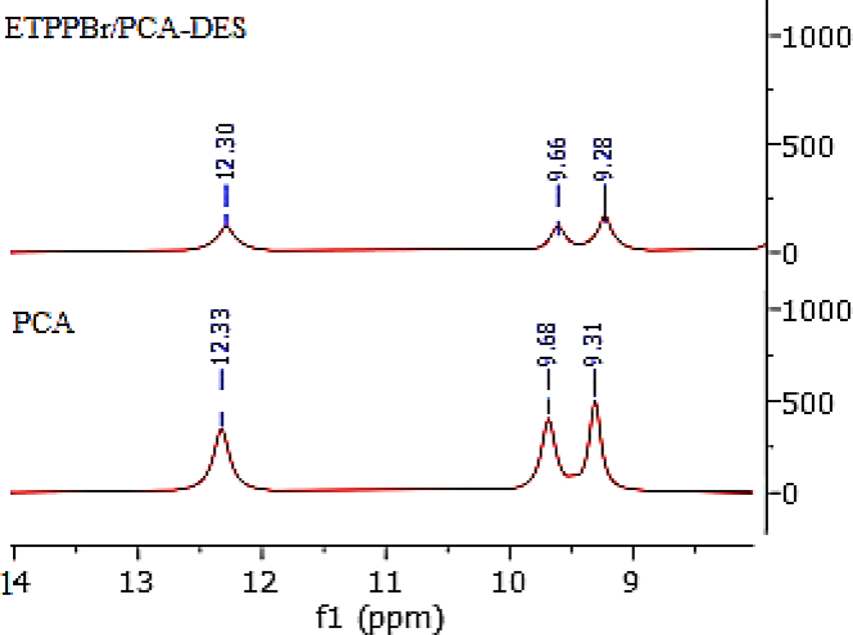
The ^1^H NMR spectra of PCA and DES.

Fig. 6 shows the comparison of the ^1^H NMR spectra of PCA and DES. As can be seen, the peaks related to the hydrogen of two hydroxy groups at position 3 and 4 and the hydrogen of the –COOH in DES have an integral decrease compared to PCA with a slight shift. The weakness of these two peaks is due to the intramolecular hydrogen bond and DES formation. Shifts of these peaks compared to the starting material is clear evidence for the DES formation.^36^

#### 3.1.4. Characterization of the ETPPBr/PCA-DES by TGA-DTA

**Fig. 7:**
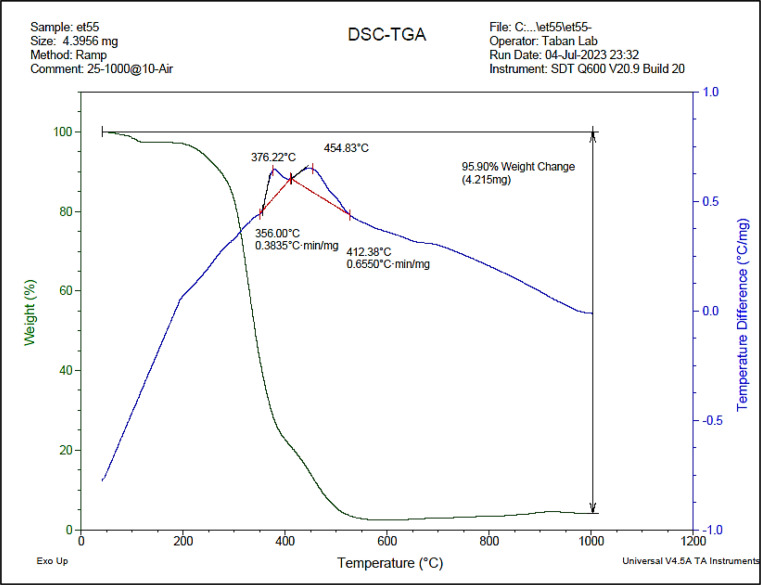
The TGA-DTA pattern of the novel DES.

To investigate the stability and thermal behavior of ETPPBr/PCA-DES, the TGA-DTA analysis was performed (Fig. 7). The curve apparently shows five weight losses at about 200, 356, 376, 454 and 700 °C which are probably related to the removal of water, organic solvents, acidic compound, breakdown of the hydrogen bond and ionic bonds, and decomposition of molecules, respectively. These results indicate the high thermal stability of ETPPBr/PCA-DES.

#### 3.1.5. Characterization of ETPPBr/PCA-DES by densitometer

DESs usually have a density of 1.0 to 1.35 g mL^−1^, so a certain weight of the novel DES was mixed with a certain volume of water and its density was calculated using the relevant formula,^37^ which is about 1.252 g mL^−1^.

### 3.2. Optimization of the reaction conditions for the synthesis of a6

The effect of different solvents, temperatures, and amounts of the DES catalyst was investigated on model reaction (reaction of phenanthrene-9,10-dione, aniline, benzaldehyde, and ammonium acetate) to optimize the reaction conditions.

First, the model reaction was carried out with different amounts of the DES catalyst which 0.5 g = 1.0 mmole of the catalyst had the best yield (Table 1).

**Table 1:** Optimization of the DES amount at 80 °C, in 10 min

Entry	DES^a^ (g, mmole)	Yield (%)
1	0.78–1.5	81
**2**	**0.50–1**	**92**
3	0.26–0.5	60
4	0.13–0.25	56

a 0.5 g of the DES catalyst is about 1.0 mmole (MW of Ph_3_PEt–Br = 371 and MW of PCA = 154, so the MW of DES = 371 + 154 = 525. As a result, 0.5 g of DES is equal to 0.5 ÷ 525 × 1000 = 0.95 ≈ 1.0 mmole.

Then, the model reaction was conducted in different temperatures which the temperature of 80 °C had the best efficiency (Table 2).

**Table 2:** Optimization of temperature by DES (0.5 g) in 10 min

Entry	Temp. (°C)	Yield (%)
1	60	50
**2**	**80**	**92**
3	100	91
4	120	95

Finally, the model reaction was performed in ethanol, water, ethyl acetate, *n*-hexane, and the DES conditions which the DES conditions showed to be the best (Table 3).

**Table 3:** Optimization of solvent by DES (0.5 g), in 10 min

Entry	Temp. (°C)	Solvent	Yield (%)
1	Reflux	Ethanol	50
2	Reflux	Water	10
4	Reflux	Ethyl acetate	33
5	Reflux	*n*-Hexane	—
**6**	**80**	**Solvent free**	**92**

Overall, we concluded that the best condition was found to be the 1 : 1 : 1 : 1.5 molar ratio of phenanthrene-9,10-dione, aniline, benzaldehyde, and ammonium acetate with 0.5 g (1.0 mmol) of the DES catalyst at 80 °C in 10 minutes.

### 3.3. Synthesis of diverse a1–a12

Based on the results obtained from the optimized conditions (synthesis of a6), a1–a12 were synthesized from the reaction of phenanthrene-9,10-dione, aromatic amine, aromatic aldehyde, and ammonium acetate at 80 °C in 0.5 g of the DES catalyst with high yields and appropriate times (Table 4).

**Table 4:** Synthesis of a1–a12 by the DES catalyst

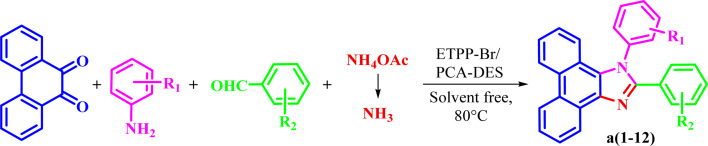								
Entry	R_1_	R_2_	Product	Time (min)	Yield (%)	M.P. °C (found, lit.) ref.	TON^a^	TOF^b^
1	H	3-NO_2_	a1	5	97	206–208, NEW	97	19.4
2	4-Cl	4-iso-Pr	a2	5	89	234–236, NEW	89	17.8
3	4-Cl	3-NO_2_	a3	13	91	182–185, NEW	91	7
4	4-Cl	4-Me	a4	10	93	181–182 NEW	93	9.3
5	H	4-iso-Pr	a5	5	90	188–189, NEW	90	18
6	H	H	a6	10	92	200–202, 206 (ref. 32)	92	9.2
7	4-Cl	3,4-diOMe	a7	7	88	184–186 (ref. 33)	88	12.5
8	H	4-Cl	a8	10	97	234–235, 233 (ref. 34)	97	9.7
9	H	4-Me	a9	6	98	200–201, 186–189 (ref. 35)	98	16.3
10	4-Cl	H	a10	15	75	220–221, 227–228 (ref. 34)	75	5
11	4-Cl	4-OMe	a11	17	89	240–244, 176–178 (ref. 35)	89	5.2
12	4-Cl	4-*N*,*N*-diMe	a12	16	90	239–240, 224–226 (ref. 33)	90	5.6

a TON (turn over number): mole of aldehyde converted per mole of the catalyst.

b TOF (turn over frequency): TOF = TON ÷ time.

**Scheme 3:**
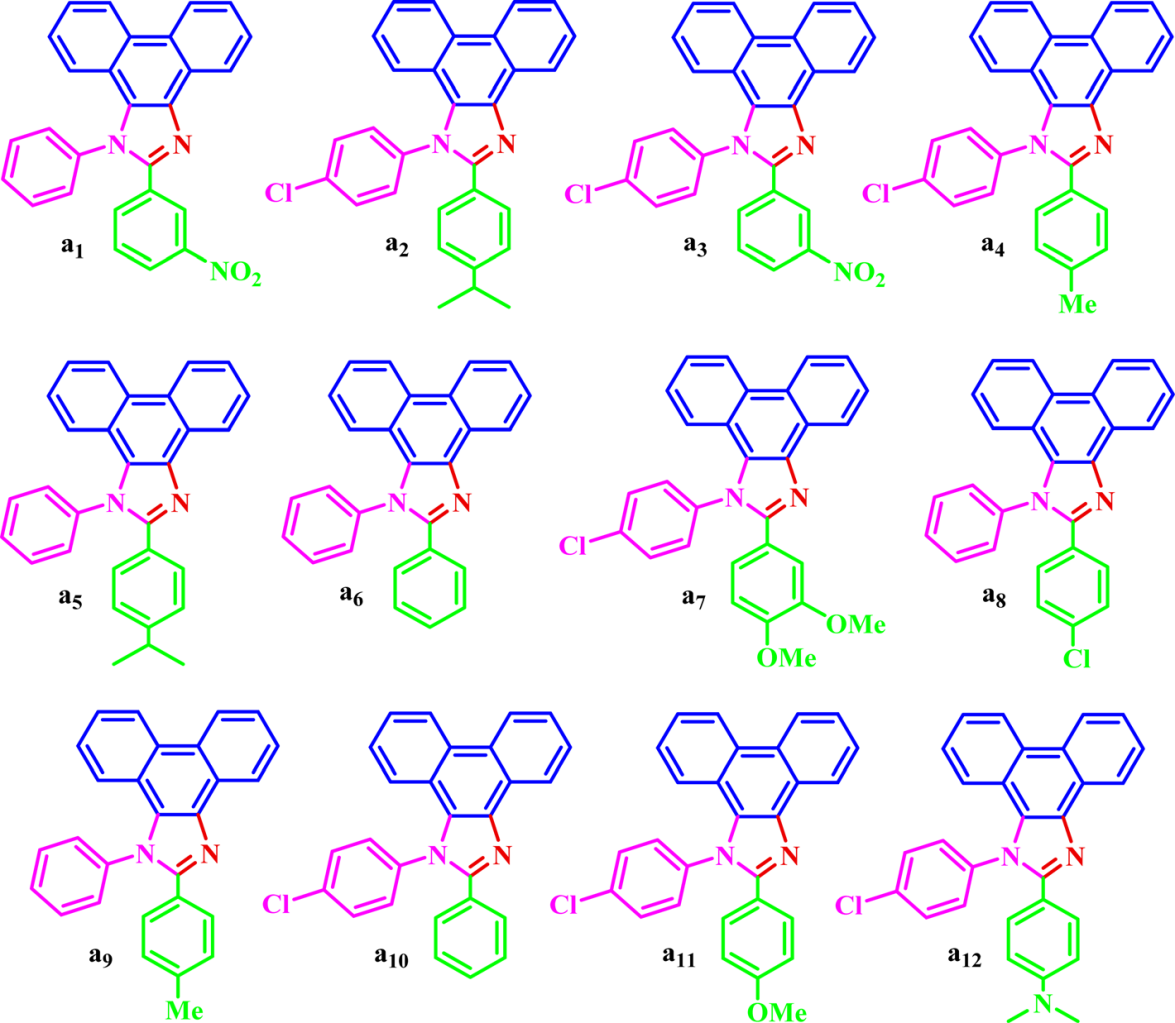
The structural formula of a1–a12.

The corresponding structural formula of a1–a12 is given below (Scheme 3).

### 3.4. A proposed mechanism for the synthesis of a1–a12 is presented below

**Scheme 4:**
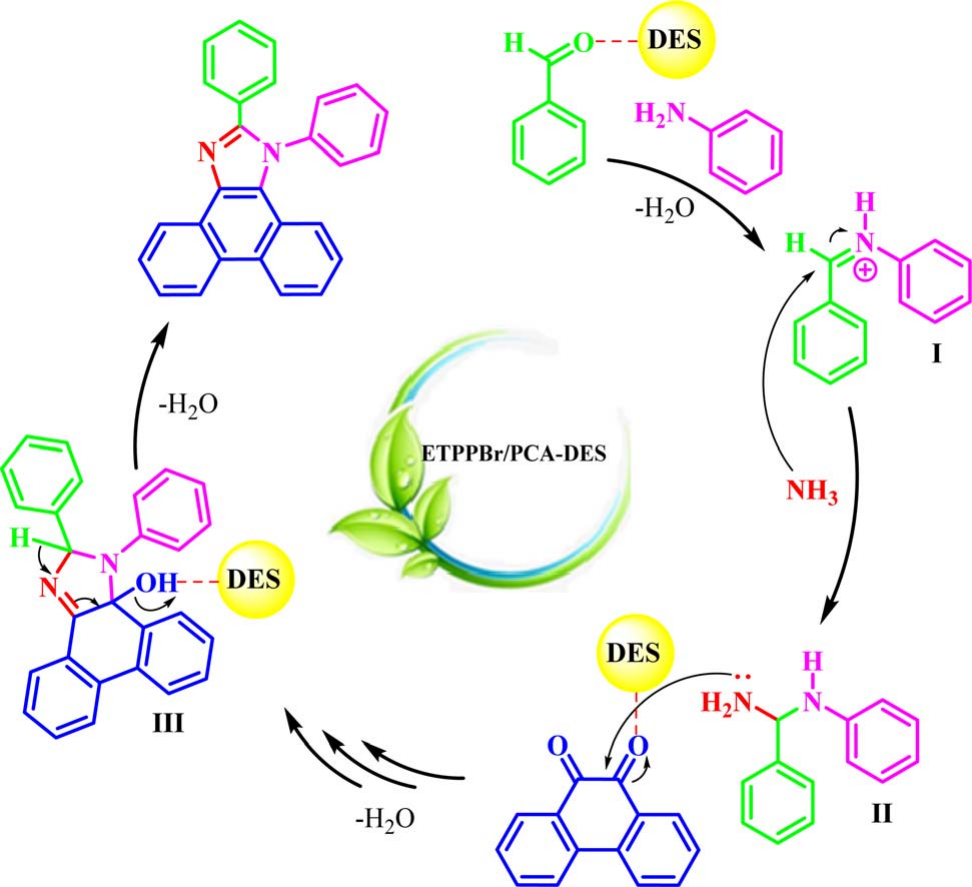
Proposed mechanism for the synthesis of a1–a12 by the DES catalyst.

The possible mechanism for the reaction is shown in Scheme 4.

First, aniline attacks to the DES-activated aldehyde to give the imine intermediate I.

Next, NH_3_ (from H_4_NOAc) will attack to intermediate I to give the intermediate II. Then, the intermediate II attacks to the DES-activated phenanthrene-9,10-dione with the subsequent internal cyclization and removing of water to from the intermediate III. Removing of water from intermediate III produces the final product.

### 3.5. Reusability of the novel DES catalyst

**Fig. 8:**
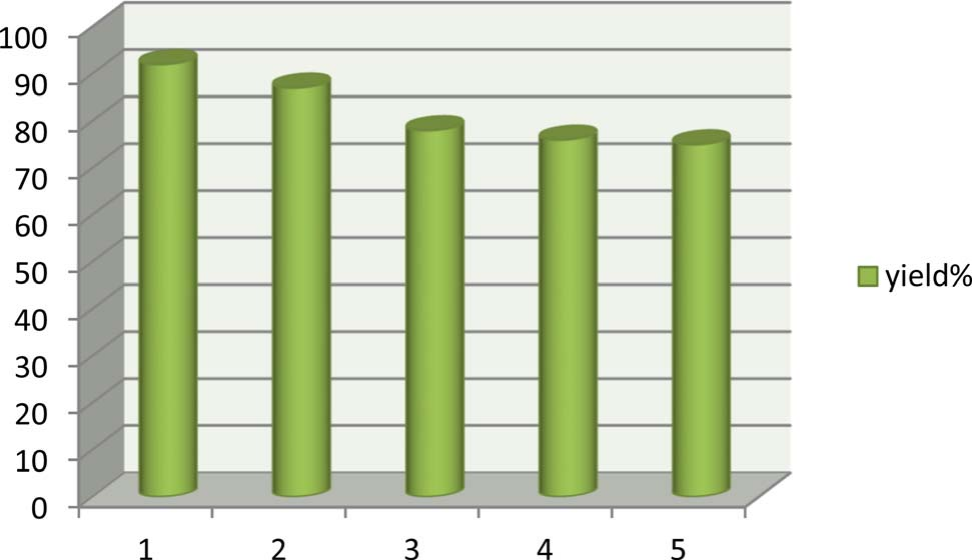
Reusability of the DES catalyst.

After completion of the reaction, the mixture was diluted with water and chloroform and shaked well. Then, the aqueous layer containing the DES catalyst was separated from the organic layer by simple liquid–liquid extraction, dried to remove water, and reused in successive runs. Results show no significant loss of catalytic activity (92, 87, 78, 76 and 75% respectively), confirming the stability of the DES catalyst (Fig. 8).

### 3.6. Comparison of the catalyst activities

Table 5 shows the comparison of different methods for the synthesis of a1–a12 indicating the advantage of our proposed procedure over other methods.

**Table 5:** Comparison of DES with other catalysts

Entry	Catalyst	Condition	Time (min)	Yield (%)	Ref.
1	CTSA	Solvent free/90 °C	32	86	35
2	MPS	MeOH + H_2_O/rt	300	86.5	33
3	K_5_CoW_12_O_40_·3H_2_O	140 °C	120	88.6	38
4	SBPPSA	Solvent free/140 °C	60	90	39
5	MCM-41	AcOH/reflux	32	82	40
6	H_3_PW_12_O_40_/Fe_3_O_4_@Si	EtOH/reflux	60	95	41
7	MCM-41-nPr-NHSO_3_H	Solvent free/130 °C	76	95.5	42
8	[Bmim]Br	Solvent free/140 °C	120	90	43
9	ETPPBr/PCA-DES	Solvent free/80 °C	10	92	This work

## 4. Conclusion

In conclusion, a novel DES was prepared by a mixture (molar ratio 1 : 1) of ETPPBr and PCA, characterized with different methods and used as a capable and novel catalyst for the green synthesis of the five new a1–a5 and the seven known 1,2,4,5-tetrasubstituted imidazoles a6–a12 in solvent-free conditions, in very short reaction time, high yields, and easy recycling and separation of the DES catalyst.

## Data availability

All data generated or analyzed during this study are included in this published article and its ESI.†

## Conflicts of interest

The authors declare that they have no known competing financial interests or personal relationships that could have appeared to influence the work reported in this paper.

## Supplementary Material

RA-014-D4RA03302G-s001
