# Supplementary material for: The protocatechuic acid-based deep eutectic solvent-mediated green synthesis of 1,2,4,5-tetrasubstituted imidazoles
Source: RSC Adv. 2024 Jul 16;14(31):22459–69. doi: 10.1039/d4ra03302g (PMC11249645; doi:10.1039/d4ra03302g)

Electronic Supplementary Material (ESI) for RSC Advances.
This journal is © The Royal Society of Chemistry 2024

Supporting Information

(RSC Advances)

The protocatechuic acid-based deep eutectic solvent-mediated green synthesis of 1,2,4,5-tetrasubstituted imidazoles

Hadis Goudarzi, Davood Habibi*, Arezo Monem

Department of Organic Chemistry, Faculty of Chemistry and Petroleum Chemistry, Bu-Ali Sina University, Hamedan 6517838683, Iran
*Corresponding author email: davood.habibi@gmail.com (& dhabibi@basu.ac.ir), Tel: +98 81 38380922; Fax: +98 81 31408025

Content	Page
FT-IR spectrum of a1	3
^{1}H NMR spectrum of a1	3
^{13}C NMR spectrum of a1	4
Mass spectrum of a1	4
FT-IR spectrum of a2	5
^{1}H NMR spectrum of a2	5
^{13}C NMR spectrum of a2	6
Mass spectrum of a2	6
FT-IR spectrum of a3	7
^{1}H NMR spectrum of a3	7
^{13}C NMR spectrum of a3	8
Mass spectrum of a3	8
FT-IR spectrum of a4	9
^{1}H NMR spectrum of a4	9
^{13}C NMR spectrum of a4	10
Mass spectrum of a4	10
FT-IR spectrum of a5	11
^{1}H NMR spectrum of a5	11
^{13}C NMR spectrum of a5	12
Mass spectrum of a5	12
FT-IR spectrum of a6	13
FT-IR spectrum of a7	13
FT-IR spectrum of a8	13
^{1}H NMR spectrum of a8	14
FT-IR spectrum of a9	14
^{1}H NMR spectrum of a9	15
FT-IR spectrum of a10	15
FT-IR spectrum of a11	16
^{1}H NMR spectrum of a11	16
^{13}C NMR spectrum of a11	17
FT-IR spectrum of a12	17

FT-IR spectrum of 2-(3-nitrophenyl)-1-phenyl-1H-phenanthro[9,10-d]imidazole (**a1**):

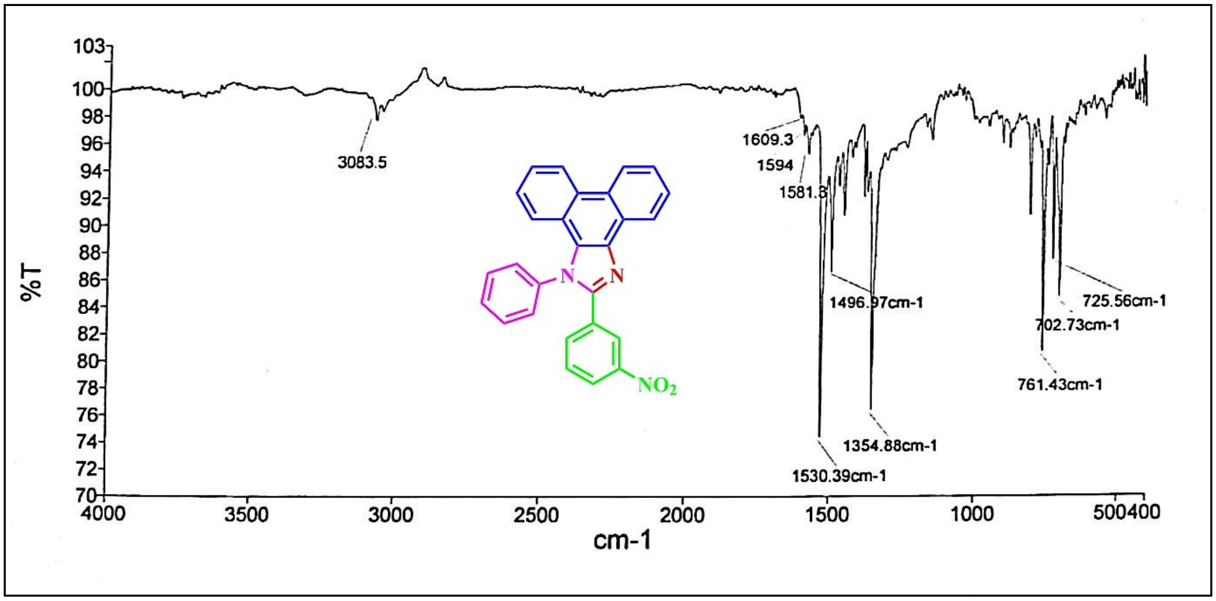

^{1}HNMR spectrum of 2-(3-nitrophenyl)-1-phenyl-1H-phenanthro[9,10-d]imidazole (**a1**):

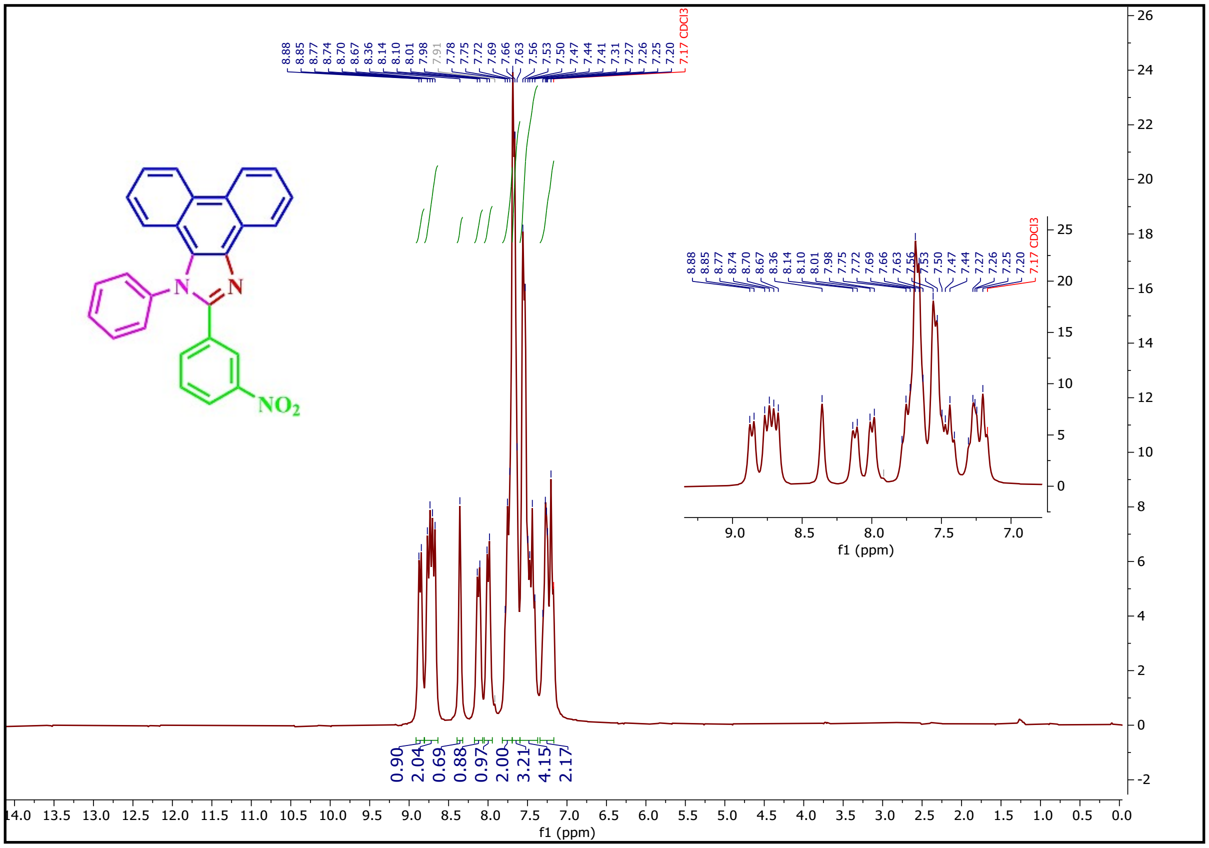

^{13}CNMR spectrum of 2-(3-nitrophenyl)-1-phenyl-1H-phenanthro[9,10-d]imidazole (**a1**):

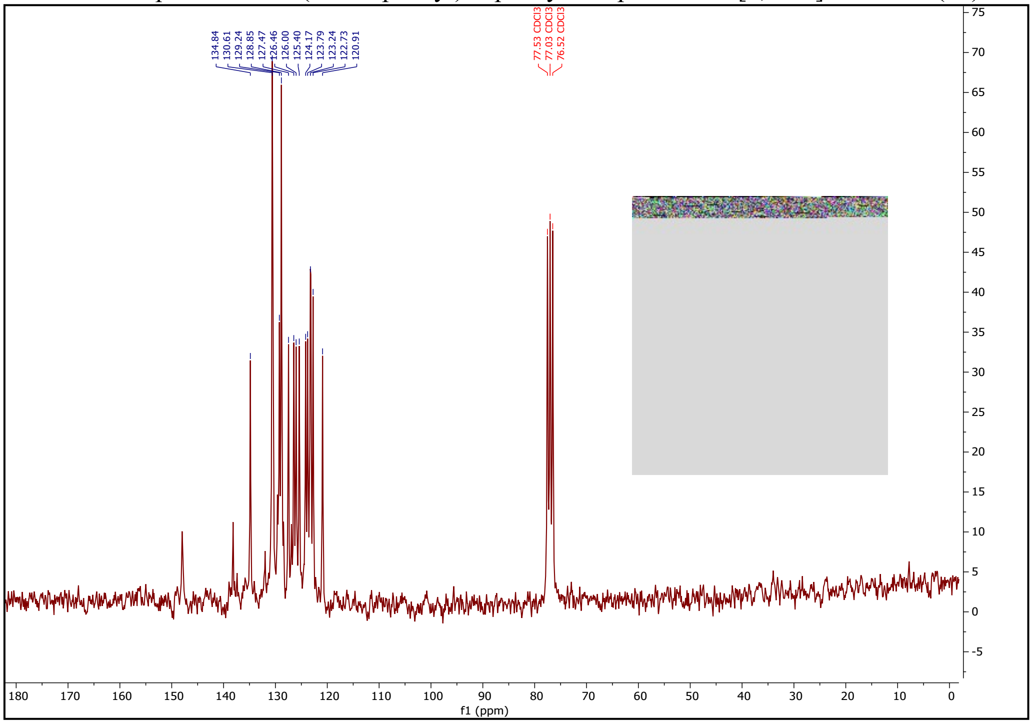

Mass spectrum of 2-(3-nitrophenyl)-1-phenyl-1H-phenanthro[9,10-d]imidazole (**a1**):

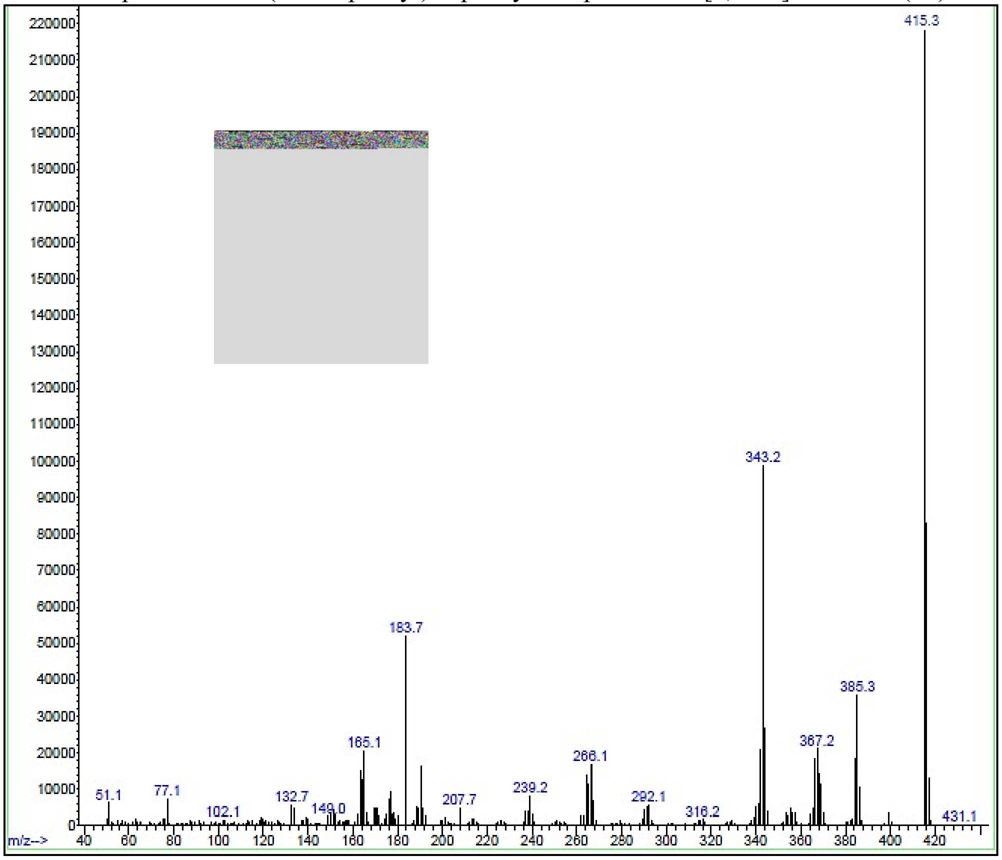

FT-IR spectrum of 1-(4-chlorophenyl)-2-(4-isopropylphenyl)-1H-phenanthro[9,10-d]imidazole (**a2**):

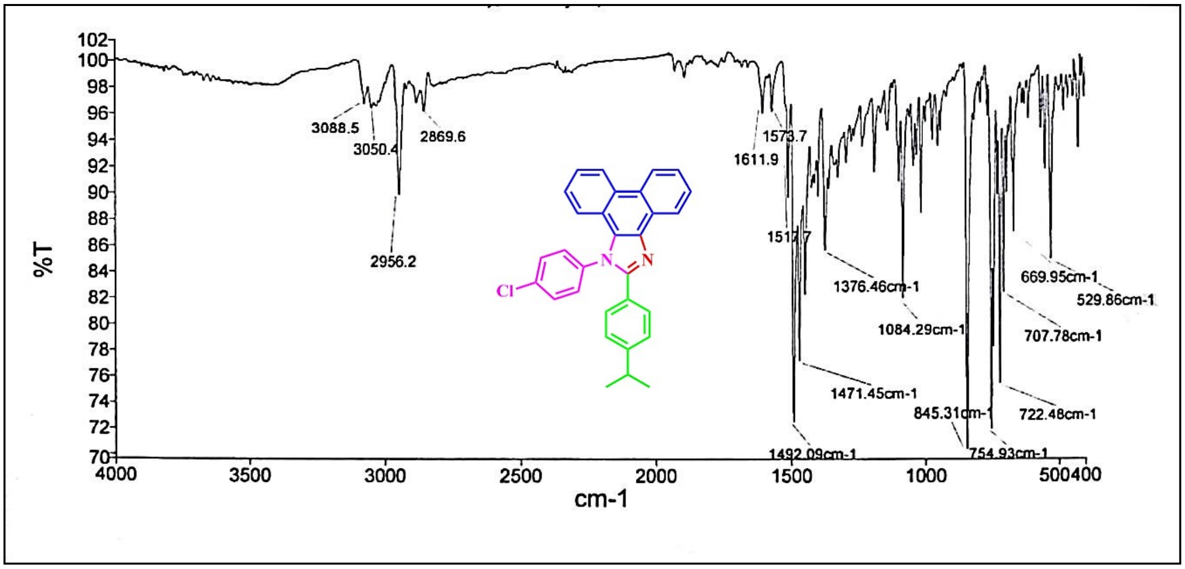

¹HNMR spectrum of 1-(4-chlorophenyl)-2-(4-isopropylphenyl)-1H-phenanthro[9,10-d]imidazole (**a2**):

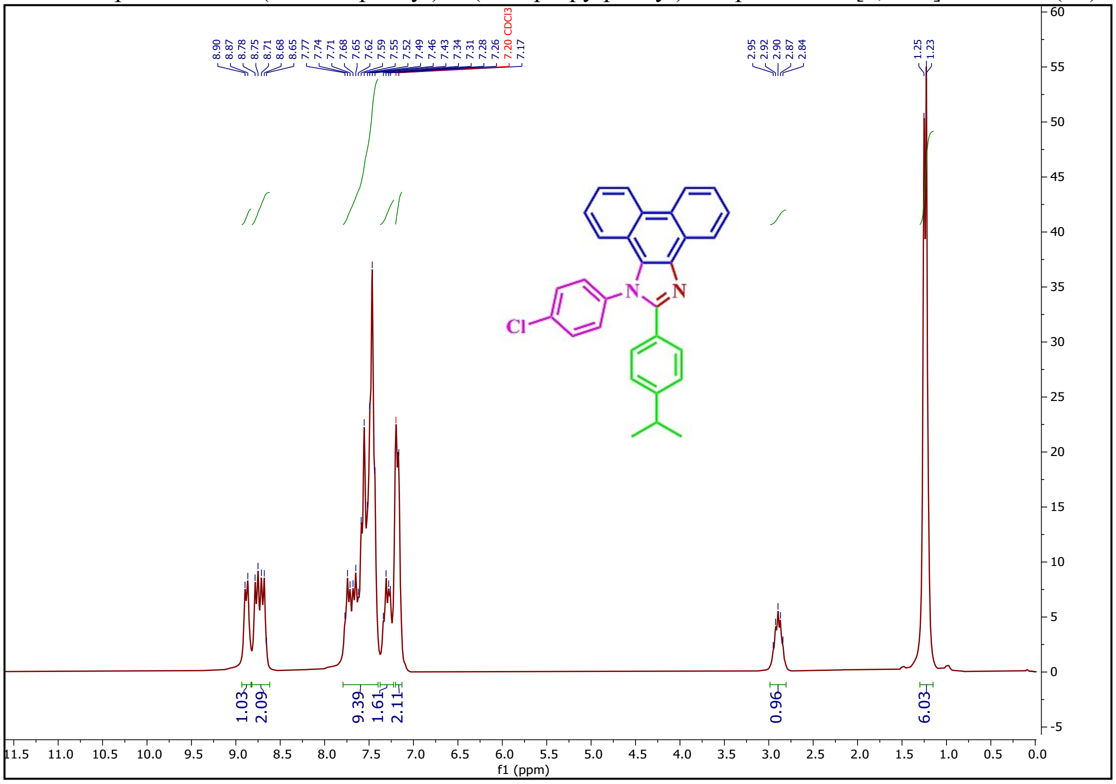

^{13}CNMR spectrum of 1-(4-chlorophenyl)-2-(4-isopropylphenyl)-1H-phenanthro[9,10-d]imidazole (**a2**):

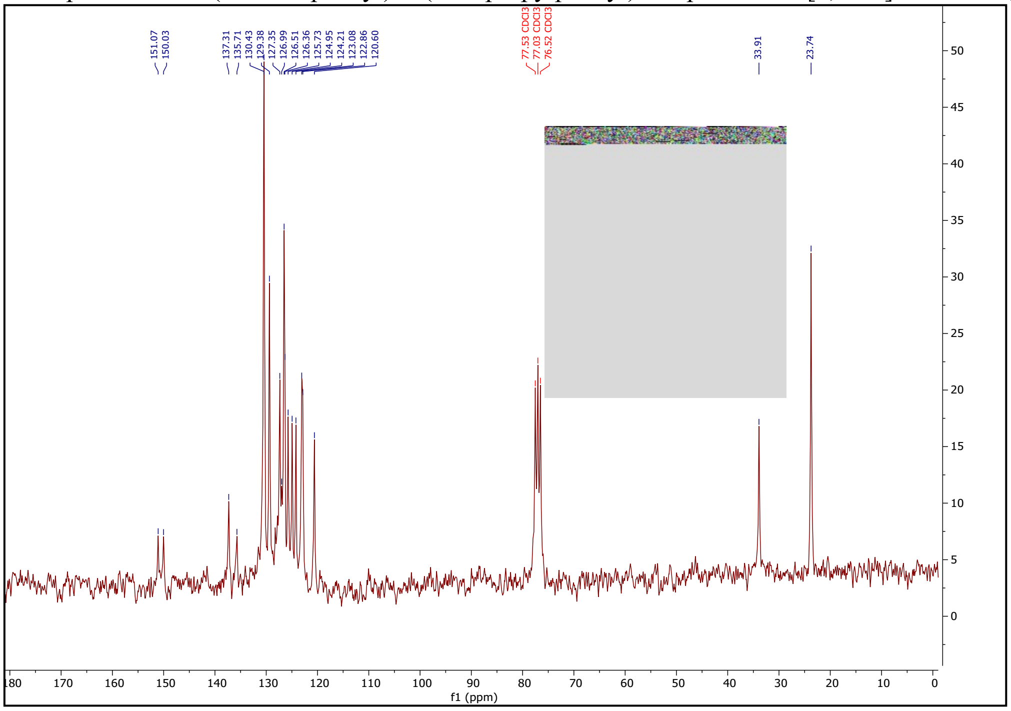

Mass spectrum of 1-(4-chlorophenyl)-2-(4-isopropylphenyl)-1H-phenanthro[9,10-d]imidazole (**a2**):

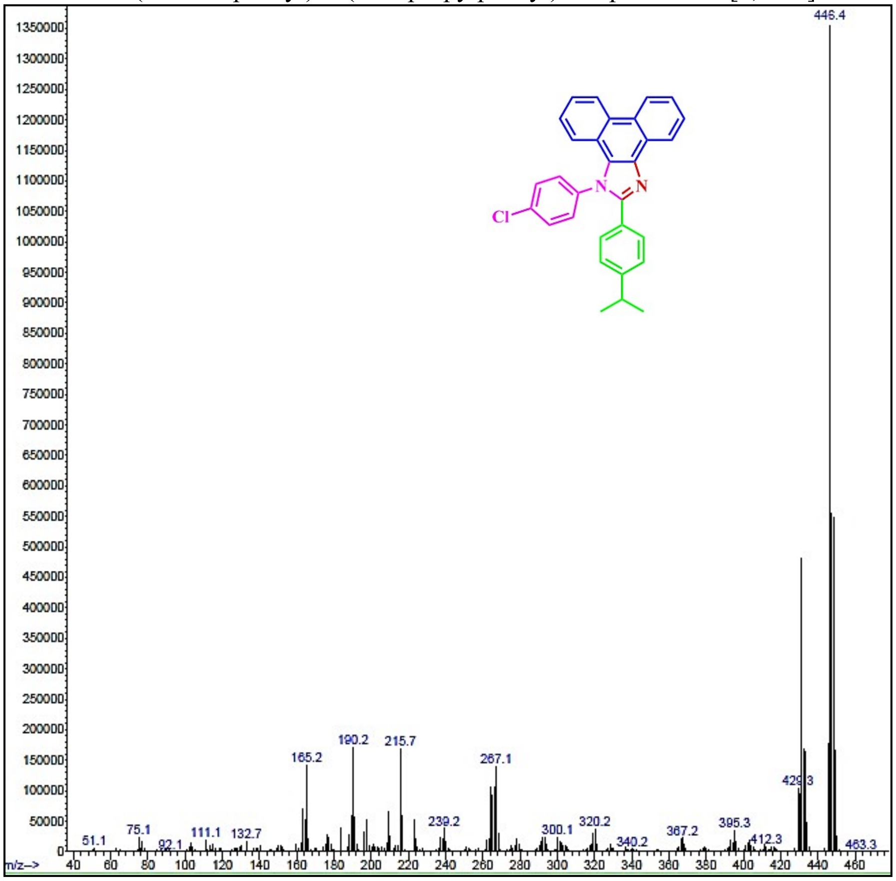

FT-IR spectrum of 1-(4-chlorophenyl)-2-(3-nitrophenyl)-1H-phenanthro[9,10-d]imidazole (**a3**):

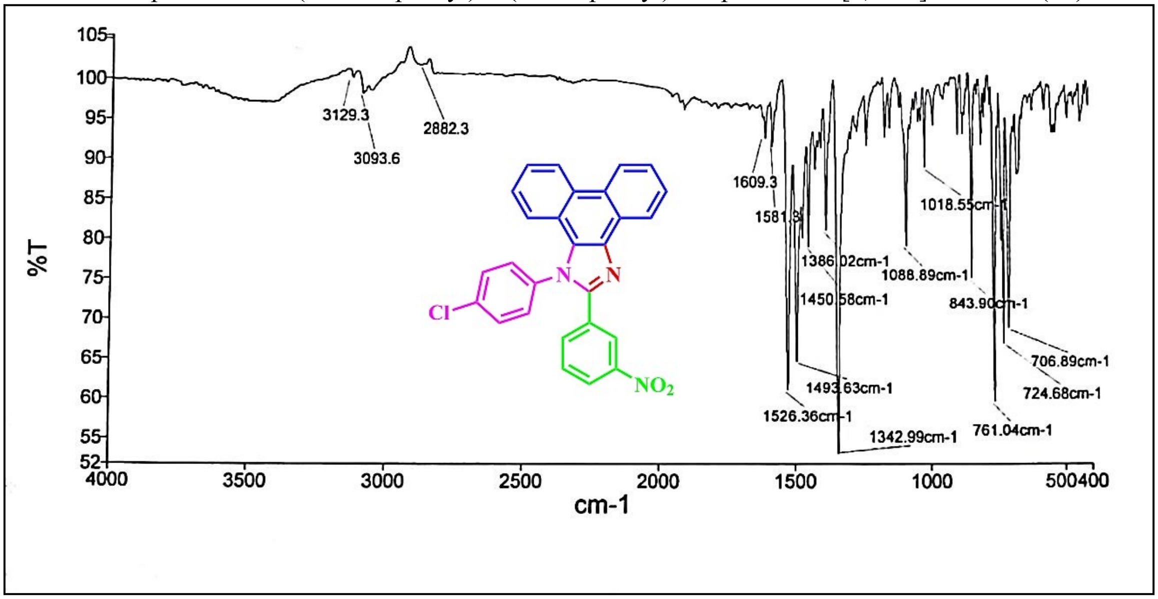

^{1}HNMR spectrum of 1-(4-chlorophenyl)-2-(3-nitrophenyl)-1H-phenanthro[9,10-d]imidazole (**a3**):

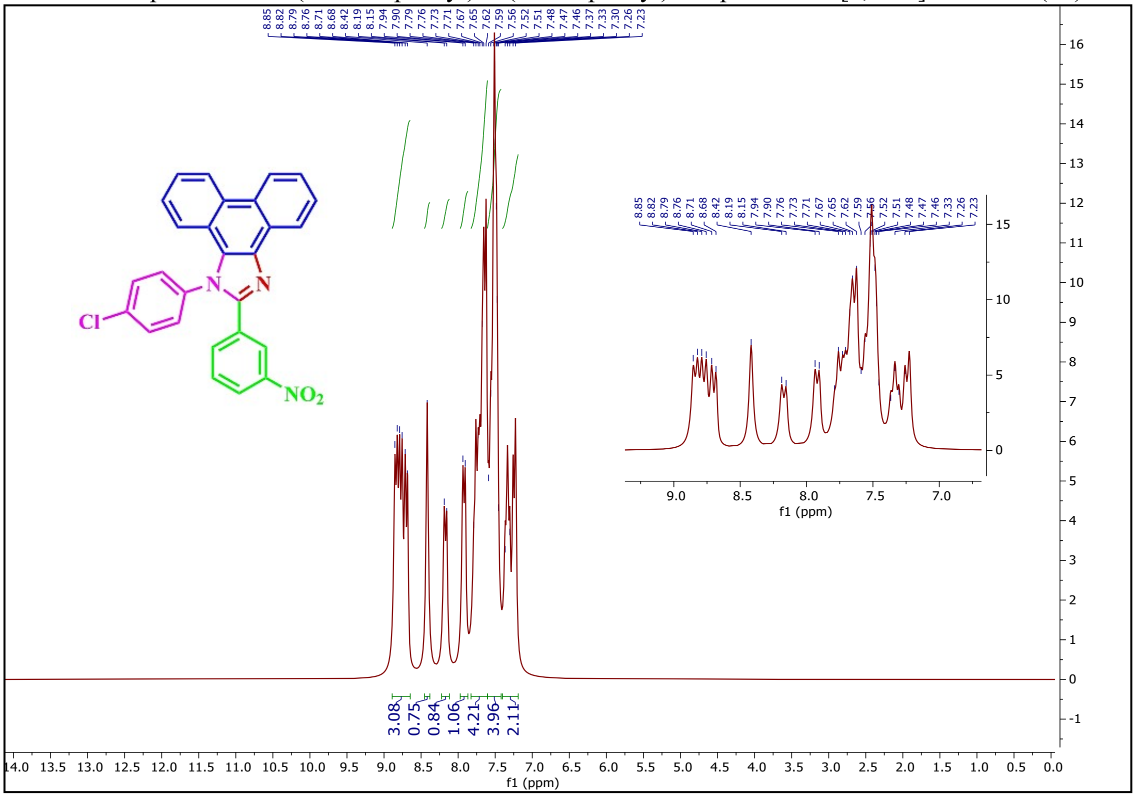

^{13}CNMR spectrum of 1-(4-chlorophenyl)-2-(3-nitrophenyl)-1H-phenanthro[9,10-d]imidazole (**a3**):

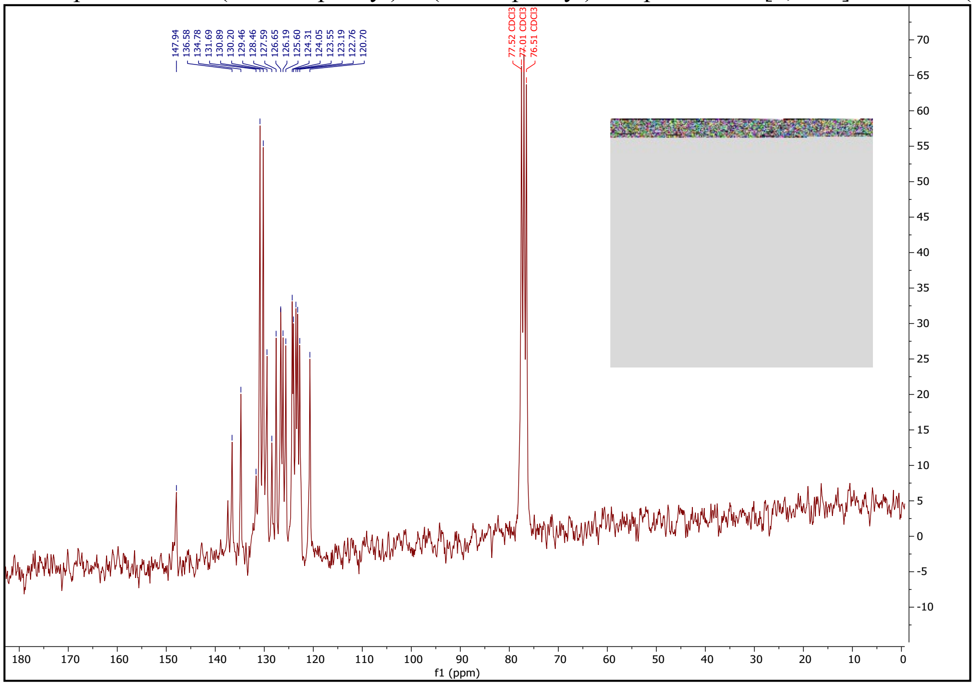

Mass spectrum of 1-(4-chlorophenyl)-2-(3-nitrophenyl)-1H-phenanthro[9,10-d]imidazole (**a3**):

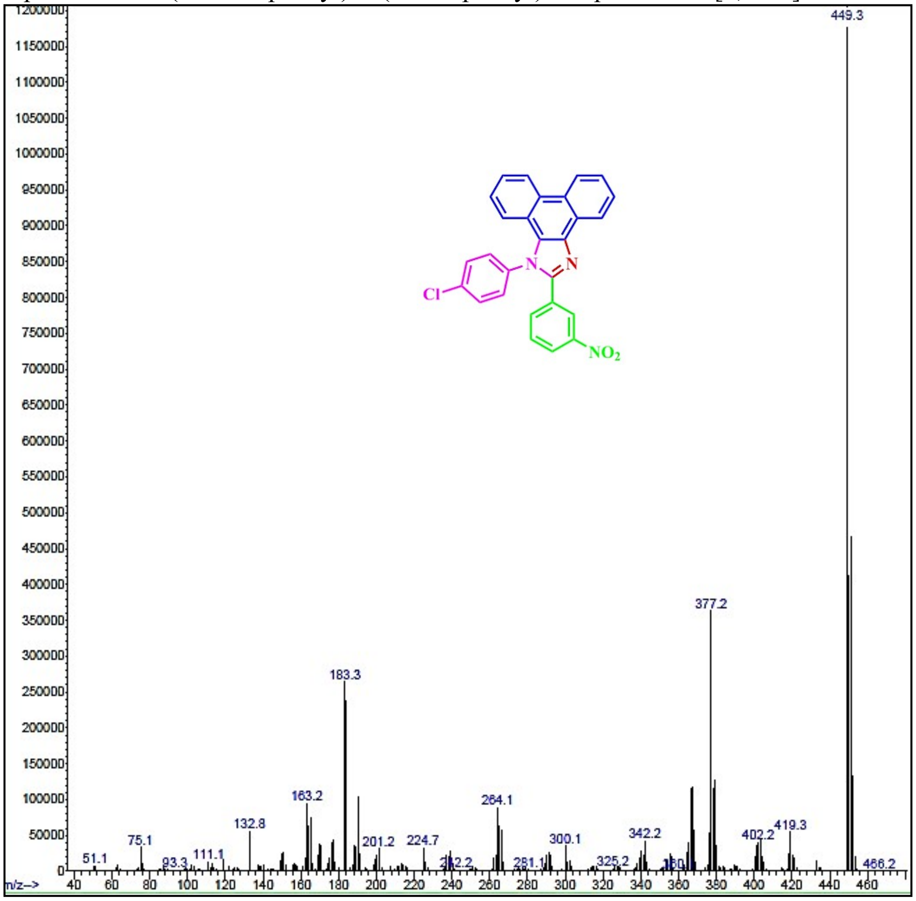

FT-IR spectrum of 1-(4-chlorophenyl)-2-(p-tolyl)-1H-phenanthro[9,10-d]imidazole (**a4**):

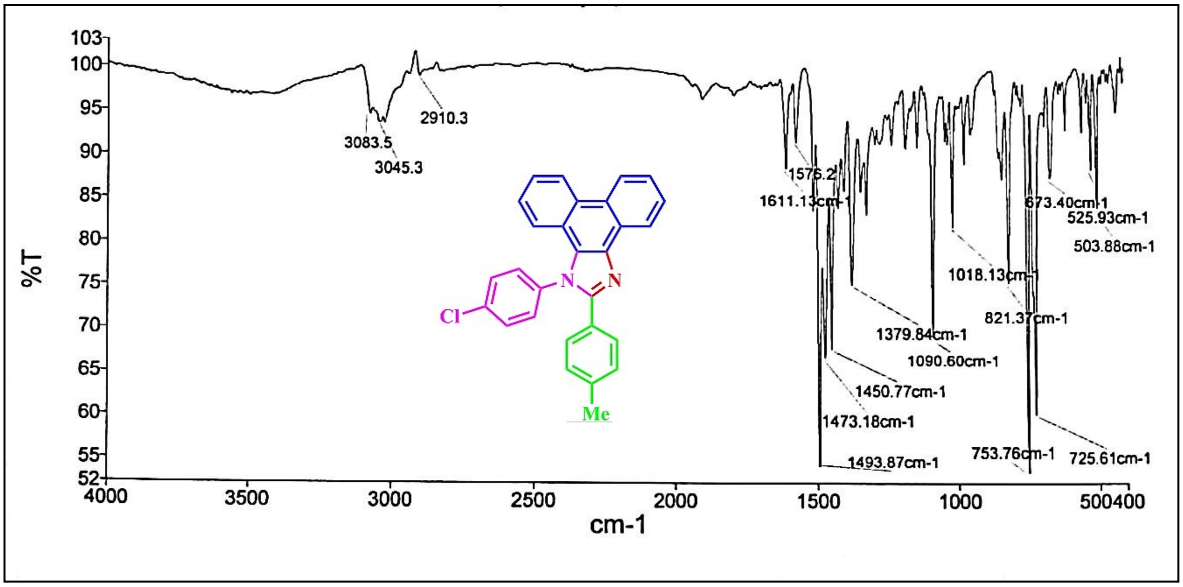

^{1}HNMR spectrum of 1-(4-chlorophenyl)-2-(p-tolyl)-1H-phenanthro[9,10-d]imidazole (**a4**):

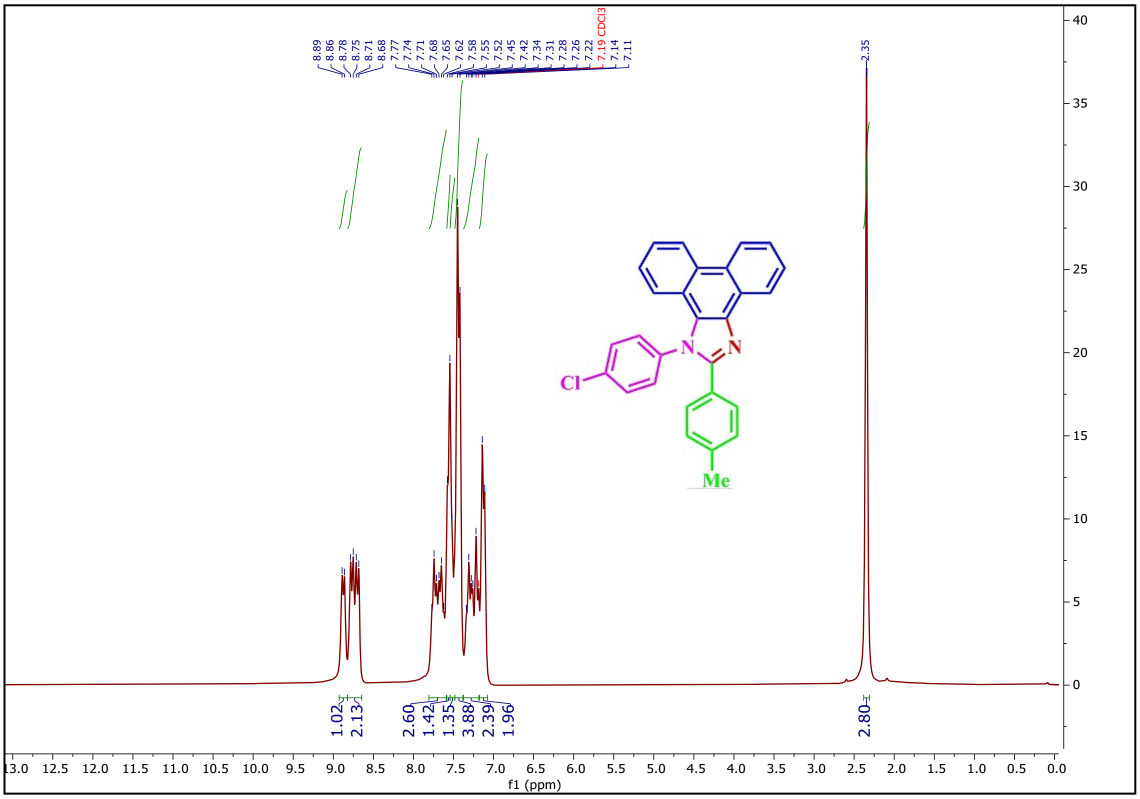

^{13}CNMR spectrum of 1-(4-chlorophenyl)-2-(p-tolyl)-1H-phenanthro[9,10-d]imidazole (**a4**):

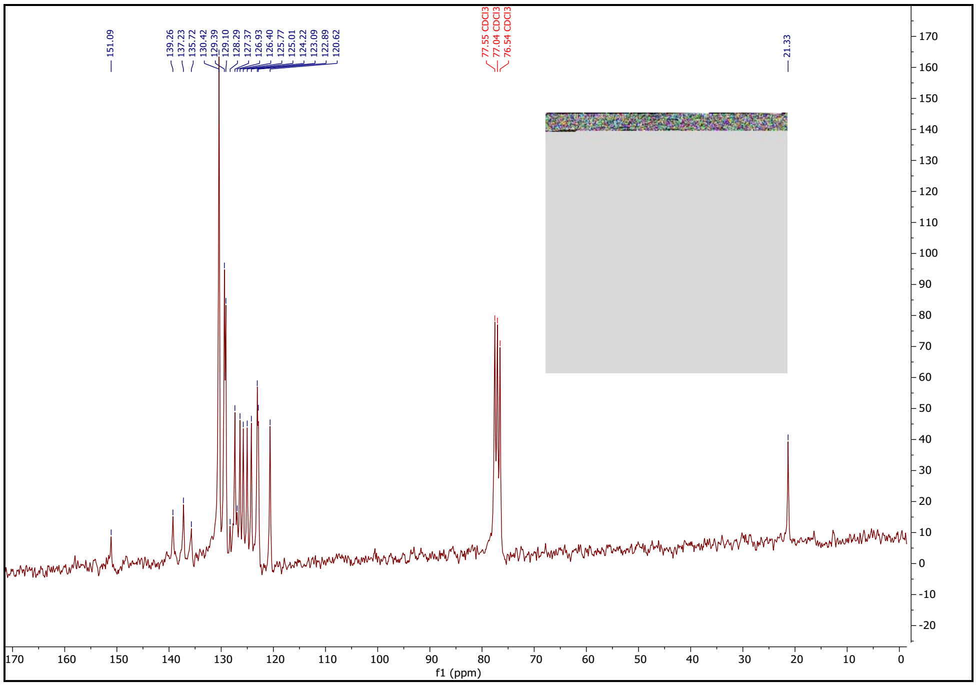

Mass spectrum of 1-(4-chlorophenyl)-2-(p-tolyl)-1H-phenanthro[9,10-d]imidazole (**a4**):

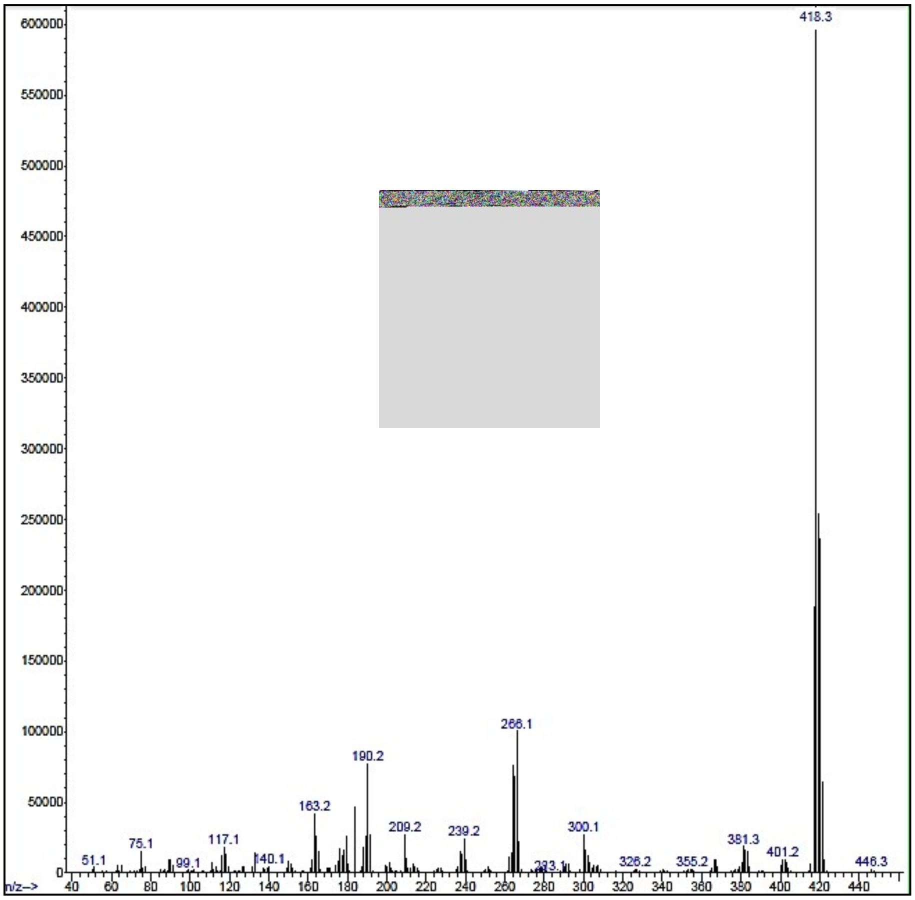

FT-IR spectrum of 2-(4-isopropylphenyl)-1-phenyl-1H-phenanthro[9,10-d]imidazole (**a5**):

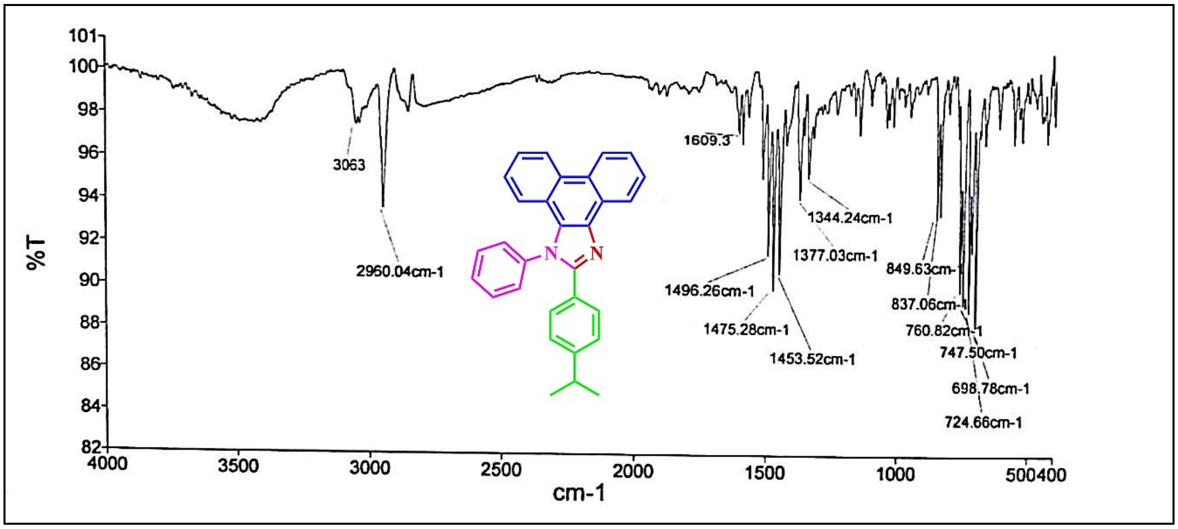

^{1}HNMR spectrum of 2-(4-isopropylphenyl)-1-phenyl-1H-phenanthro[9,10-d]imidazole (**a5**):

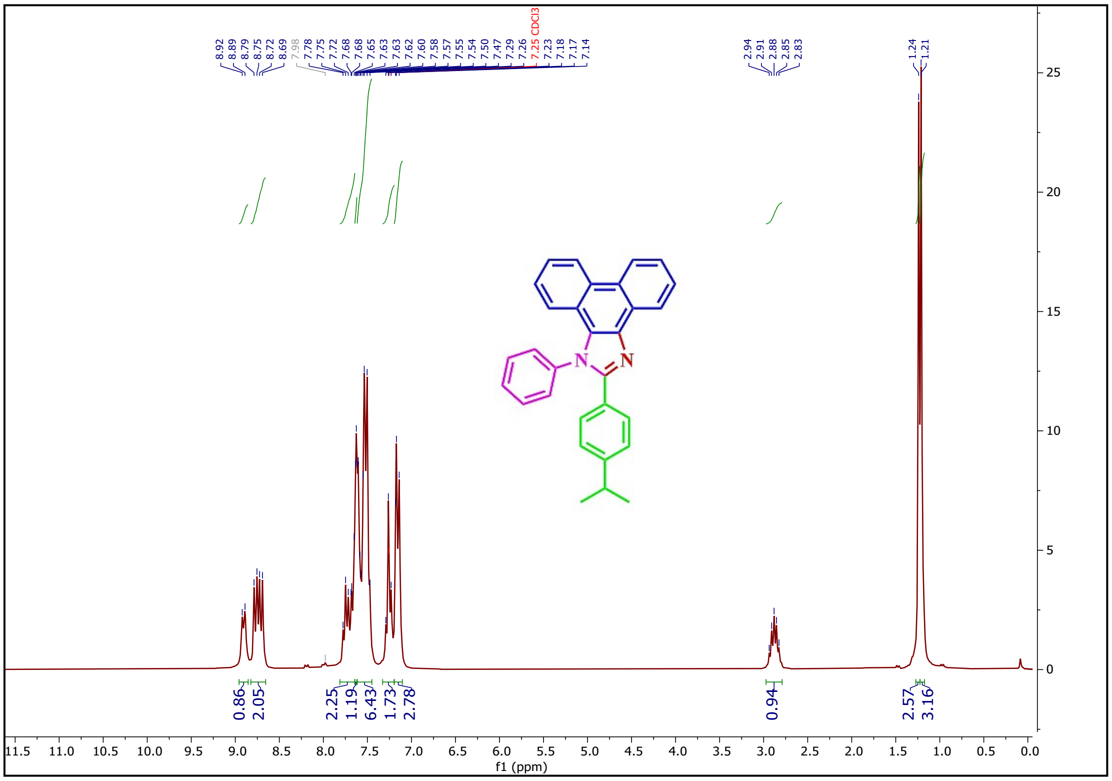

^{13}CNMR spectrum of 2-(4-isopropylphenyl)-1-phenyl-1H-phenanthro[9,10-d]imidazole (**a5**):

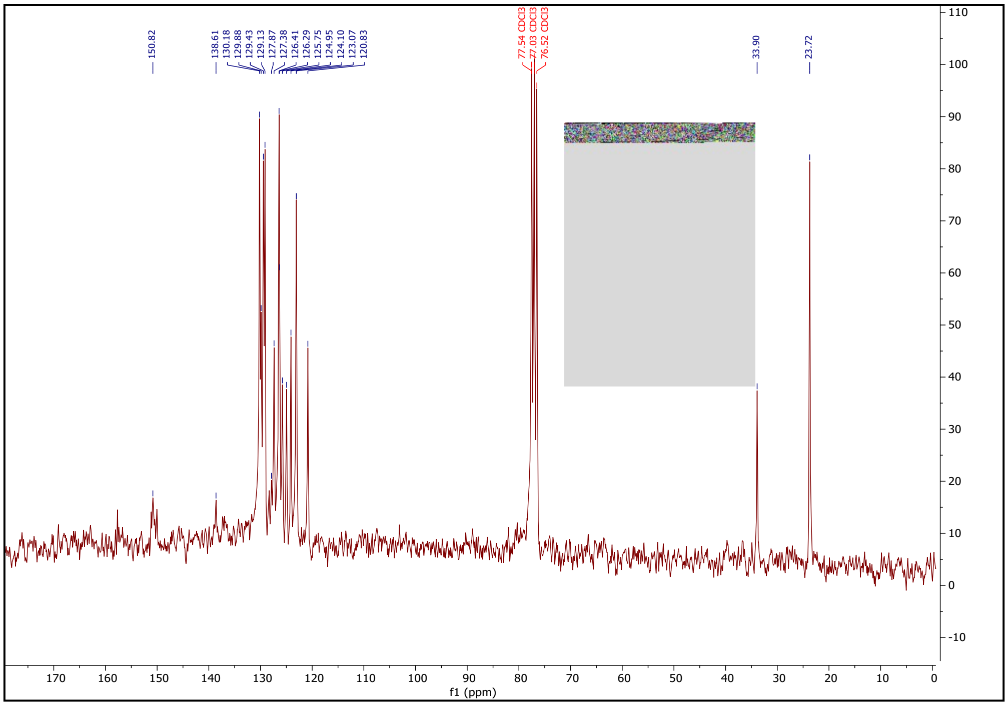

Mass spectrum of 2-(4-isopropylphenyl)-1-phenyl-1H-phenanthro[9,10-d]imidazole (**a5**):

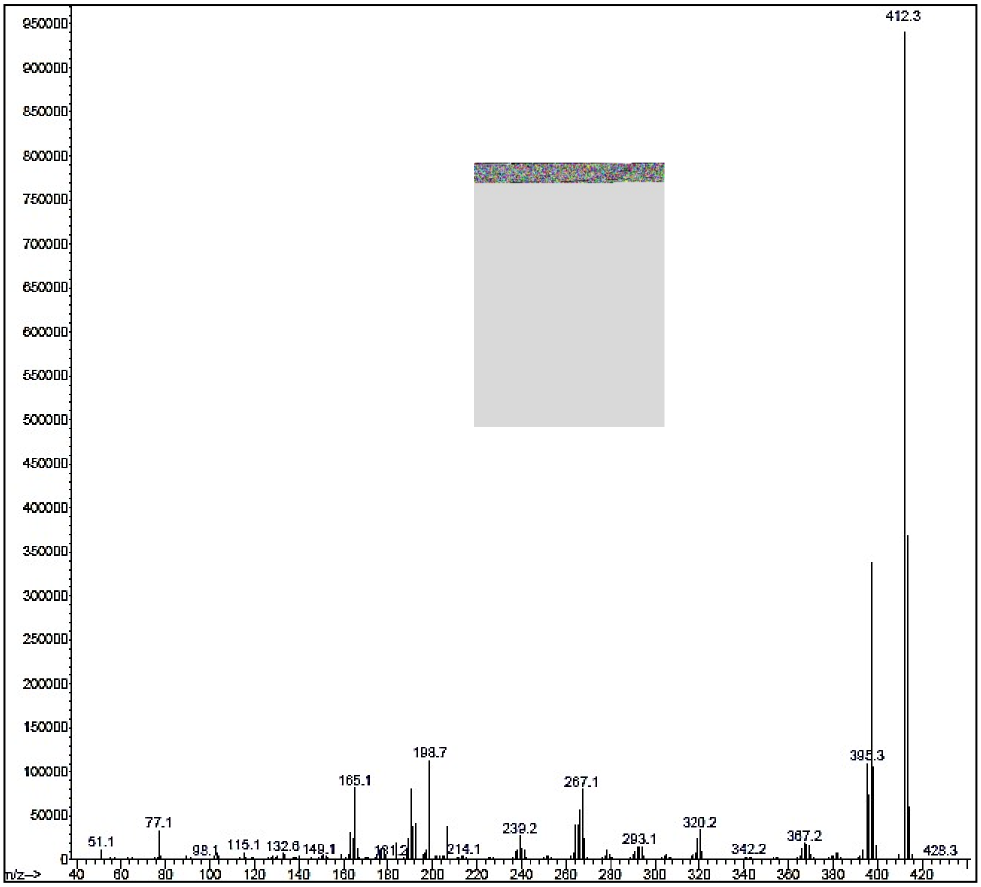

FT-IR spectrum of 1-(4-chlorophenyl)-2-(3,4-dimethoxyphenyl)-1H-phenanthro[9,10-d]imidazole (**a6**):

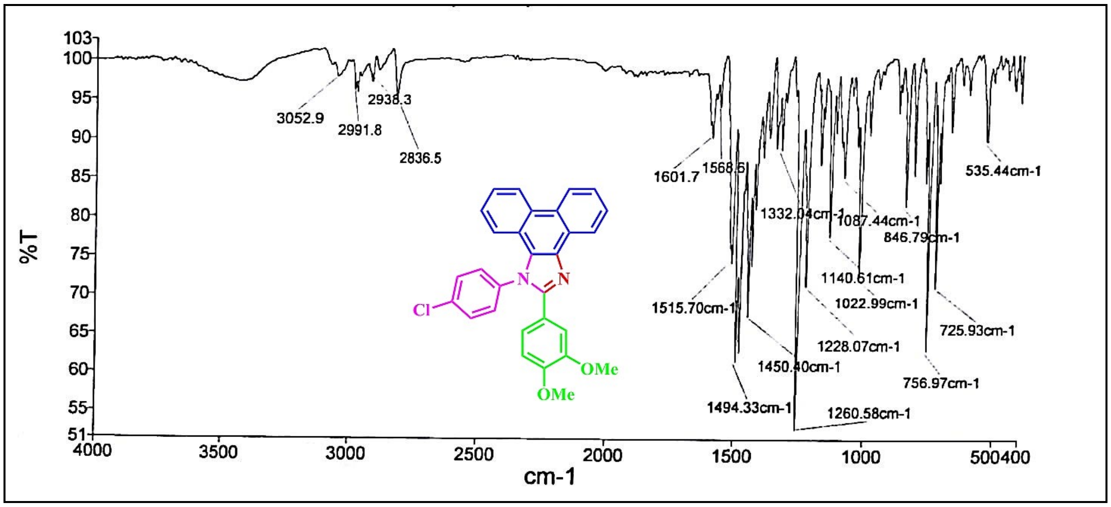

FT-IR spectrum of 1,2-diphenyl-1H-phenanthro[9,10-d]imidazole (**a7**):

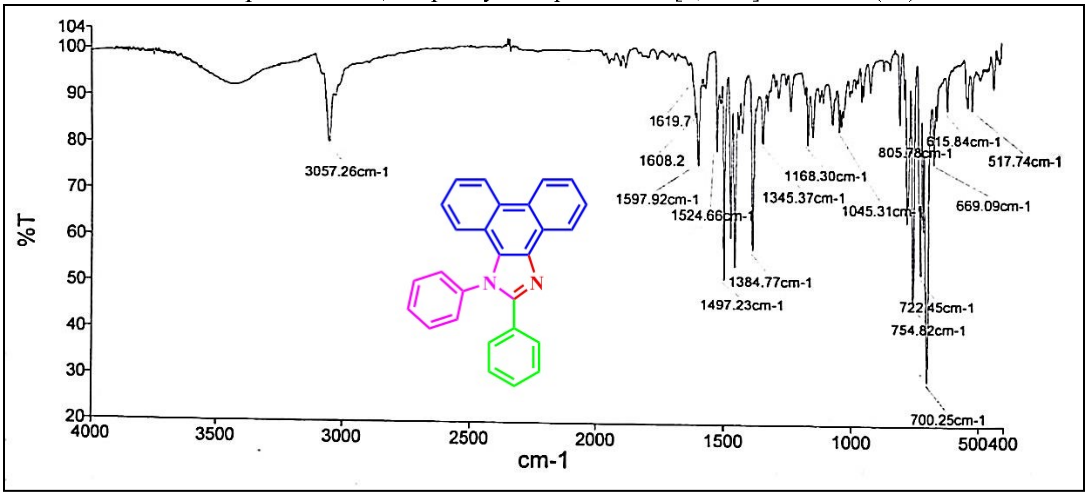

FT-IR spectrum of 2-(4-chlorophenyl)-1-phenyl-1H-phenanthro[9,10-d]imidazole (**a8**):

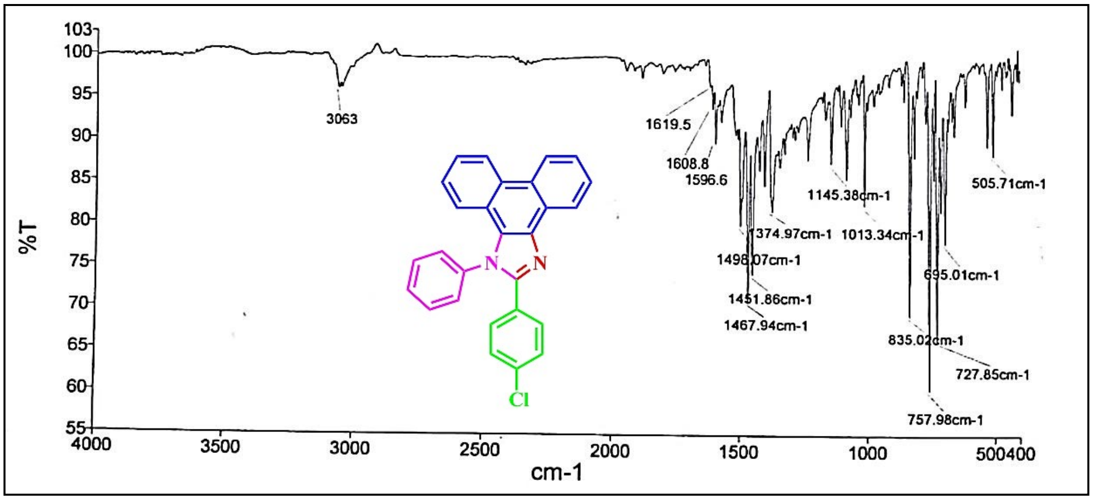

^{1}HNMR spectrum of 2-(4-chlorophenyl)-1-phenyl-1H-phenanthro[9,10-d]imidazole (**a8**):

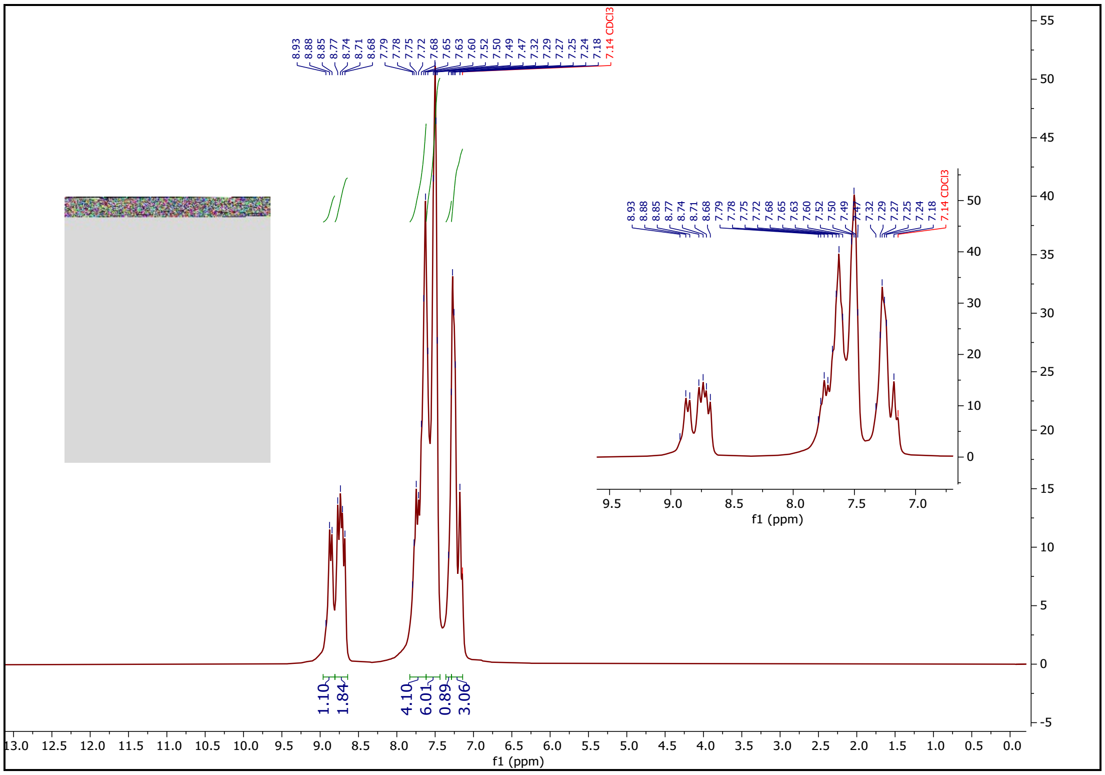

FT-IR spectrum of 1-phenyl-2-(p-tolyl)-1H-phenanthro[9,10-d]imidazole (**a9**):

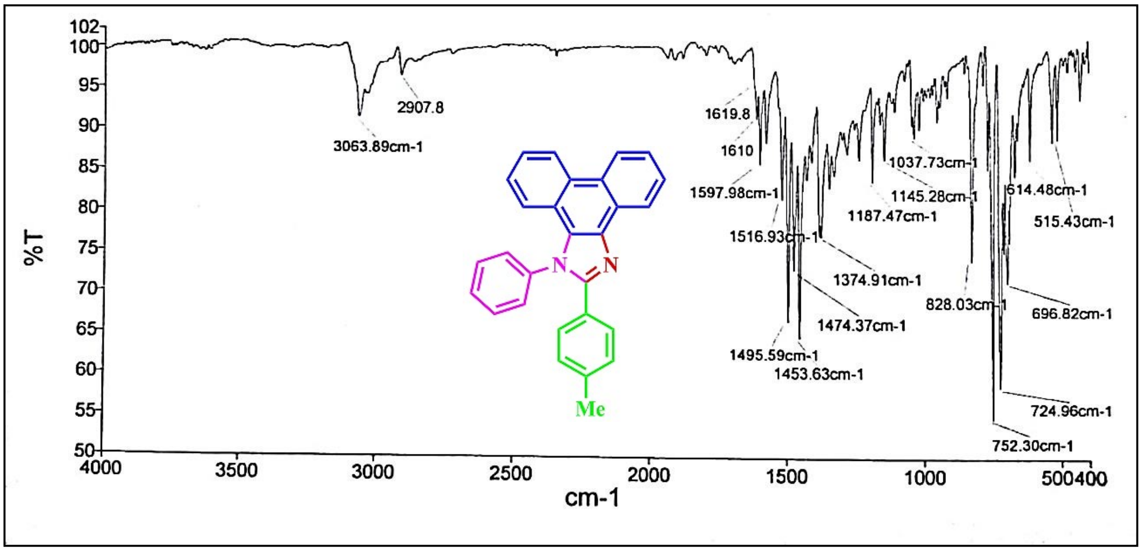

^{1}HNMR spectrum of 1-phenyl-2-(p-tolyl)-1H-phenanthro[9,10-d]imidazole (**a9**):

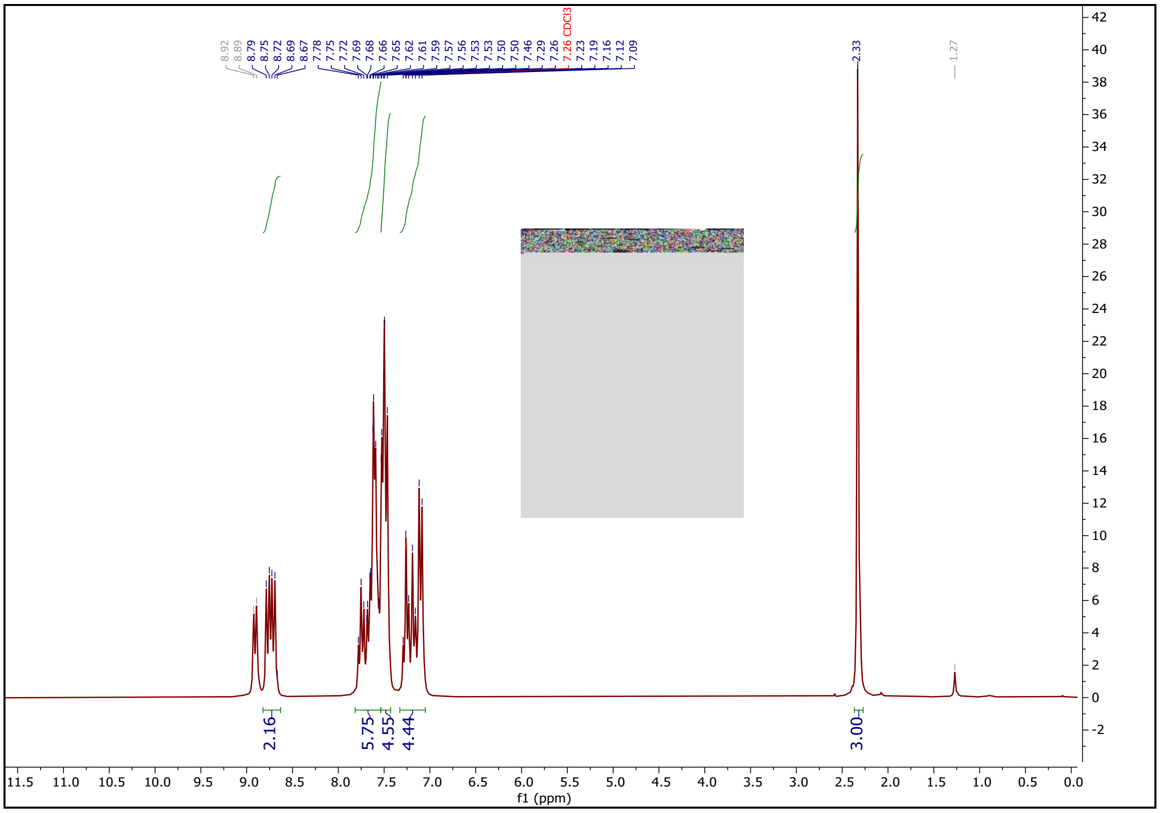

FT-IR spectrum of 1-(4-chlorophenyl)-2-phenyl-1H-phenanthro[9,10-d]imidazole (**a10**):

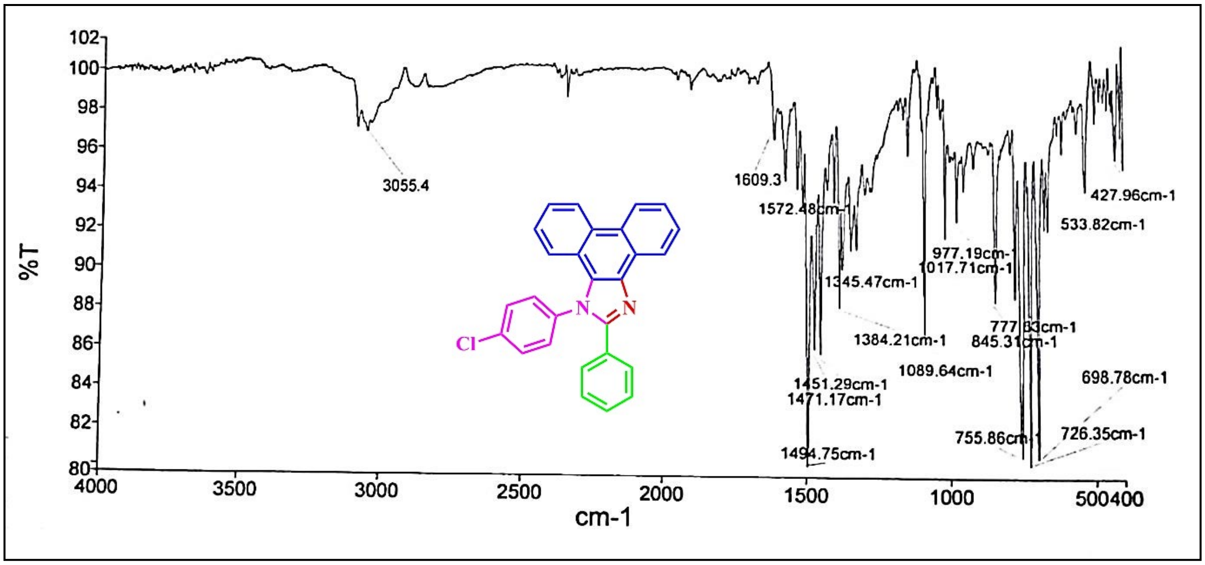

FT-IR spectrum of 1-(4-chlorophenyl)-2-(4-methoxyphenyl)-1H-phenanthro[9,10-d]imidazole (**a11**):

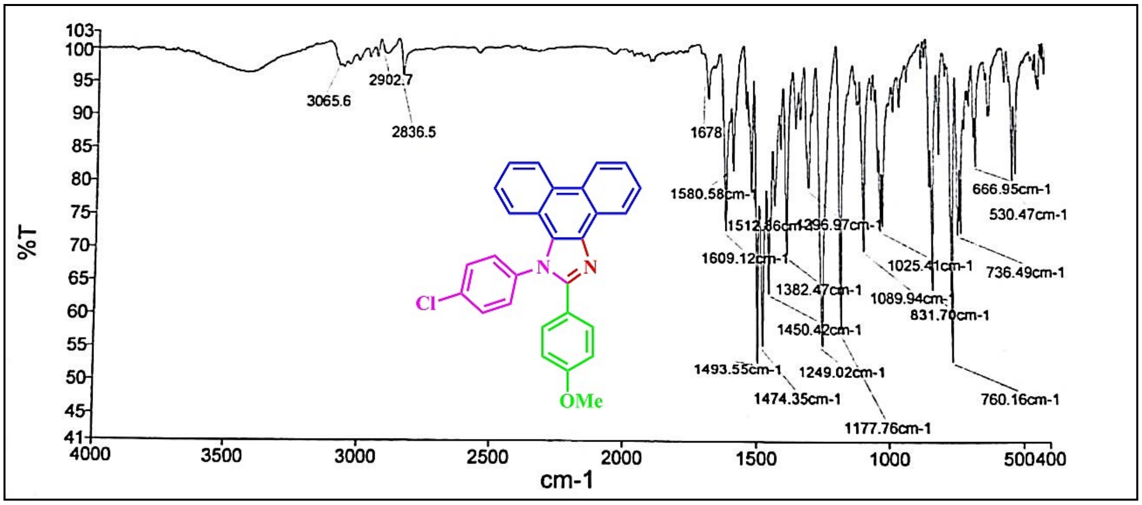

^{1}HNMR spectrum of 1-(4-chlorophenyl)-2-(4-methoxyphenyl)-1H-phenanthro[9,10-d]imidazole (**a11**):

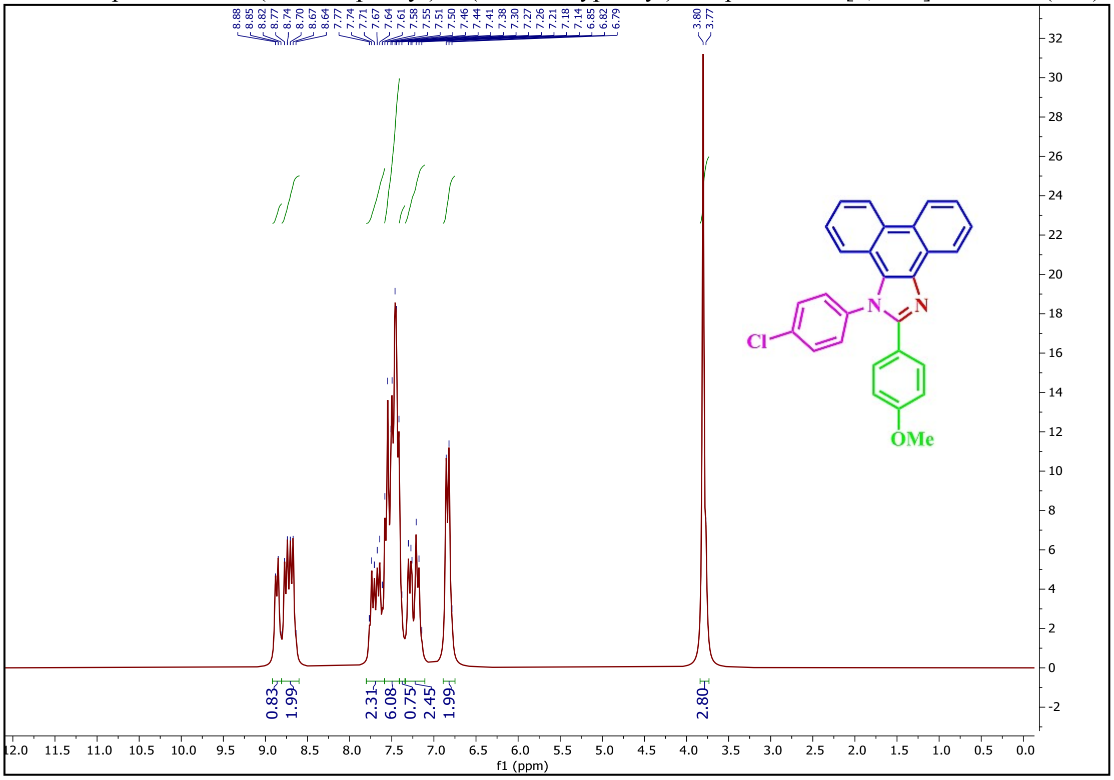

^{13}CNMR spectrum of 1-(4-chlorophenyl)-2-(4-methoxyphenyl)-1*H*-phenanthro[9,10-*d*]imidazole (**a11**):

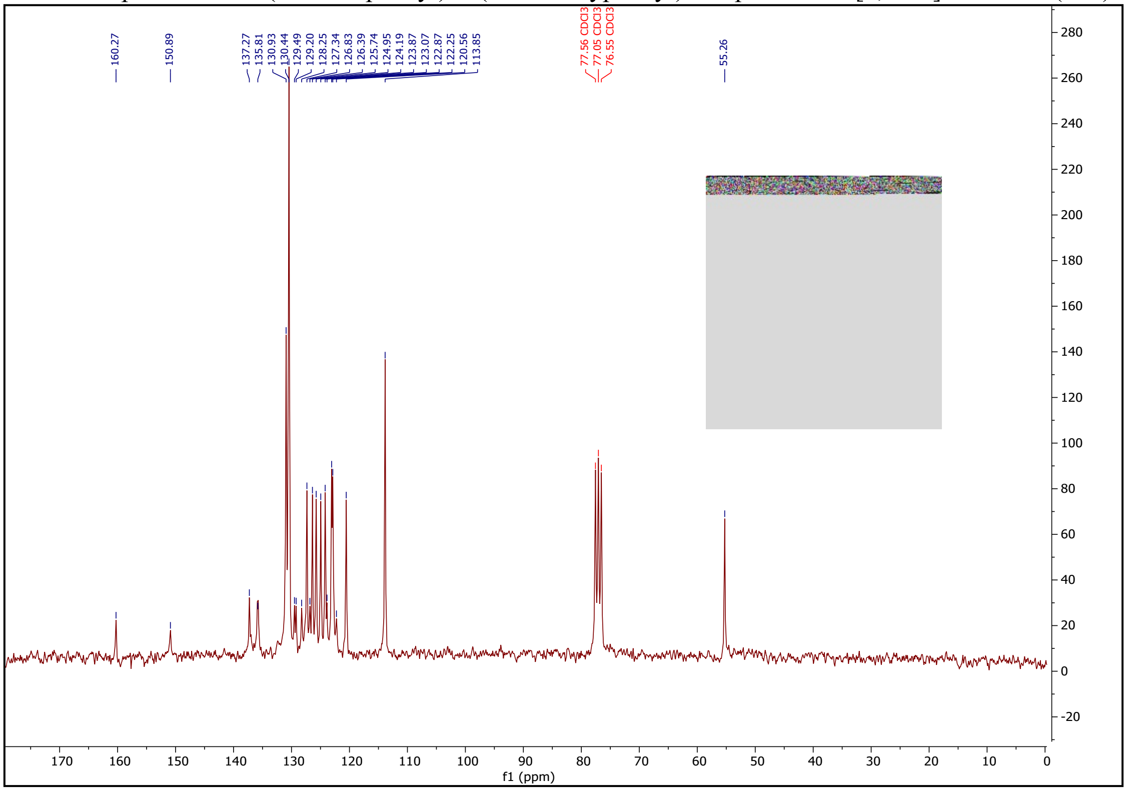

FT-IR spectrum of 4-(1-(4-chlorophenyl)-1H-phenanthro[9,10-d]imidazol-2-yl)-N,N-dimethylaniline (**a12**):

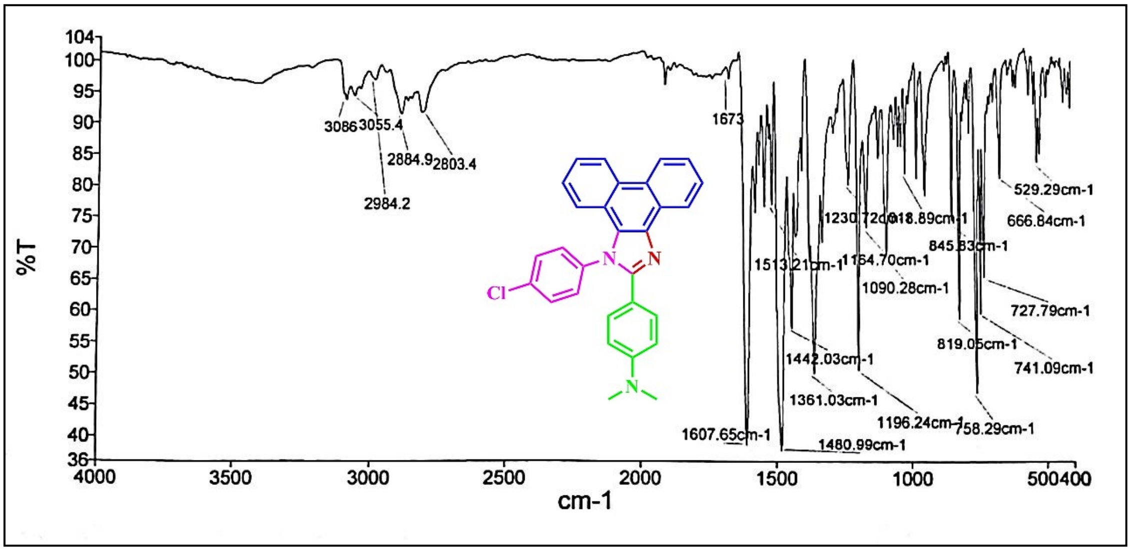

Supplement: RA-014-D4RA03302G-s001 [file RA-014-D4RA03302G-s001.pdf]
